# Exploring the Feasibility of Relapse Prevention Strategies in Interdisciplinary Multimodal Pain Therapy Programs: Qualitative Study

**DOI:** 10.2196/21545

**Published:** 2020-12-11

**Authors:** Stefan Elbers, Jan Pool, Harriët Wittink, Albère Köke, Rob Smeets

**Affiliations:** 1 Research Group Lifestyle & Health Research Centre Healthy and Sustainable Living University of Applied Sciences Utrecht Utrecht Netherlands; 2 Department of Rehabilitation Medicine Faculty of Health, Life Sciences and Medicine Maastricht University Maastricht Netherlands; 3 Centre of Expertise in Pain and Rehabilitation Adelante Hoensbroek Netherlands; 4 South University of Applied Sciences Heerlen Netherlands; 5 Centrum voor Integrale Revalidatie Eindhoven Netherlands

**Keywords:** chronic pain, interdisciplinary treatment, rehabilitation, relapse, behavior change, goal setting, pain, behavior, feasibility, strategy, treatment

## Abstract

**Background:**

Although interdisciplinary multimodal pain treatment (IMPT) programs are widely regarded as treatment of choice for patients with chronic pain, there are signs that many patients are unable to maintain their treatment gains in the long term. To facilitate the maintenance of positive treatment outcomes over time, we developed two relapse prevention strategies.

**Objective:**

The main objective of this study was to explore the feasibility of these strategies within the context of IMPT programs.

**Methods:**

We performed a feasibility study using 3 workbook prototypes containing either one or both strategies. For a period of 6 months, the workbooks were made available in two IMPT facilities. Qualitative data were collected through a focus group and semistructured interviews. We performed a thematic analysis using a deductive approach with (1) applicability to the treatment program, (2) acceptability of the workbook content, and (3) form, as predefined themes.

**Results:**

The final dataset consisted of transcripts from a focus group with health care providers and 11 telephone interviews and 2 additional in-depth interviews with patients. In general, the intervention was perceived as useful, easy to use, and in line with the treatment program. The data also include suggestions to further improve the use of both strategies, including more specific implementation guidelines, revised goal-setting procedure, and development of a mobile health version. However, several factors, including a high dropout rate and small sample size, impact the external validity of our findings.

**Conclusions:**

This study should be regarded as a first step in the process of transforming the prototype workbook into an effective intervention for clinical practice. Although these initial results indicate a favorable evaluation of both behavior regulation strategies within the workbook, this study encountered multiple barriers regarding implementation and data collection that limit the generalizability of these results. Future research efforts should specifically address the fidelity of HCPs and patients and should include clear procedures regarding recruitment and use of both relapse prevention strategies during treatment.

## Introduction

Interdisciplinary multimodal pain therapy (IMPT) programs have been developed to address the complex multifaceted nature of chronic pain. Instead of directly treating the pain itself, IMPT programs offer a comprehensive approach to target mutually interacting cognitive, behavioral, emotional, biological, and social factors to improve daily life functioning and quality of life, irrespective of pain [1-3]. Typically, these programs include an interdisciplinary team of at least three professionals from varying backgrounds that coordinate their therapeutic activities throughout the program in line with patient-centered goals and biopsychosocial treatment principles. Also, IMPT programs are generally provided within a single facility, and patients are actively engaged with their rehabilitation by means of exercises and tasks [2,4]. Although IMPT programs are often considered treatment of choice for patients with chronic pain [5], there are signs that a considerable proportion of patients are not able to maintain positive treatment outcomes over time [6-8]. This problem of relapse is not limited to IMPT; other behavioral treatments show similar trends for various patient groups, including patients following orthopedic rehabilitation [9] and patients with chronic diseases [10,11]. These results indicate that the problem of relapse may transcend disease-specific treatment.

One strategy that has been recommended to improve long-term effectiveness is to adjust the treatment program to specific individual patient characteristics, needs, and preferences [12,13]. This tailoring is specifically relevant in the domain of IMPT because these types of treatment programs seldom target one type of behavior but a complex and patient-specific cluster of health behaviors, each associated with patient-specific personal and contextual factors [14]. Moreover, in the context of IMPT, the options to realistically simulate a patient’s natural environment within the boundaries of a treatment facility are limited, which may threaten effective generalization of newly learned behaviors to patient-specific meaningful contexts [15].

To provide patients and health care providers (HCPs) with tools that could prevent relapse after successful treatment, we initiated a research project to develop a relapse prevention intervention. In iterations over a period of 18 months, patients, HCPs, pain researchers, experts on behavior change, and designers participated in co-design activities to develop two relapse prevention strategies [16]. These activities ranged from interviews with patients and HCPs to full-day cocreation sessions where ideas were developed into concrete prototypes. In the final phase of the project, the two most promising relapse prevention strategies were merged into a paper prototype intervention. This relapse prevention workbook was regarded as the optimal form to test both strategies within existing IMPT programs, given the available budget and development time. The strategies within the workbook were based on experience from practice (eg, patient interviews, stakeholder discussions, HCP feedback) and general self-regulation principles that relate to maintenance of newly learned behaviors (eg, habit formation and goal setting) [17-21]. The main goal of the workbook was to support and facilitate the transfer of individually meaningful insights and learned skills to the personal context of each patient. Treatment teams provided feedback on the workbook design to ensure that the form and content would fit existing treatment principles [22].

To determine if these strategies are eligible for further development and efficacy testing, it is important to investigate their potential in a real-world setting. According to Bowen and colleagues [23], feasibility studies are important to select promising interventions for further development and obtain specific feedback regarding factors such as usability and implementation.

To provide insight into patient and HCP evaluations regarding the relapse prevention strategies, our primary study objective was to examine the level of acceptability to stakeholders of the current workbook prototype within the context of IMPT programs. To explore how well the workbook fits within the existing treatment program, we additionally investigated the degree of applicability.

## Methods

### Study Design

To investigate the feasibility of the strategies, we conducted a qualitative study to assess patient and HCP evaluations related to acceptability and applicability of the prototype intervention within the existing treatment program. Ethical approval was granted by the local ethics committee (Medical Research Ethics Committee Zuyderland 16-N-46).

### Participants

The study was performed in two locations of the Adelante Rehabilitation Centre: Hoensbroek and Maastricht. Patients with chronic musculoskeletal pain were referred to the program by general practitioners or specialists who determined that primary care treatment was insufficient to address all existing biopsychosocial factors. Patients were eligible for treatment when their pain and pain-related disability interfered with daily life functioning to a moderate or severe extent. Patients could not participate in the rehabilitation program if they had severe or dominant psychiatric conditions, were unable to speak Dutch, were involved in ongoing legal procedures, or insisted on obtaining additional somatic diagnostic procedures. An additional criterion for location Maastricht was that patients needed to experience pain-related fear of performing certain activities in daily life that could be challenged in an in vivo exposure procedure. In this study, all patients who participated in the IMPT were eligible for inclusion.

### Treatment

The outpatient IMPT programs at both locations varied in dose and content but had a similar biopsychosocial perspective and included pain neuroscience education as well as cognitive behavioral approaches to improve physical functioning and health-related quality of life. At minimum, the treatment staff consisted of a physiatrist, physical therapist, and psychologist but could also include a social worker and occupational therapist. Both programs required active patient involvement and included regular interdisciplinary team meetings to discuss the patient’s progress. The treatment program in Hoensbroek contained multiple intervention components, including but not limited to graded activity, acceptance and commitment therapy, learning to pace (work-related activities) and set realistic goals under supervision of an occupational therapist, and functional exercise therapy such as swimming or walking under supervision of a physiotherapist. After an initial treatment phase of 3 weeks (5 days per week), a patient-specific program was created that matched dose and content with individual needs. On average, the total program was 12 weeks. The center provided accommodations for patients who were unable to commute to the center on a daily base. Location Maastricht primarily provided exposure in vivo treatment [15,24,25]. At minimum, the program was 2 weeks with 2 treatment sessions per week but could be extended up to 10 weeks depending on the complexity of the case. A typical treatment program contained 20 hours of treatment and consisted of medical education by a physiatrist (that no harm or additional injury could be inflicted by performing activities) and behavioral experiments led by a physical or occupational therapist and psychologist (half of the sessions were led by both HCPs).

### Materials

We developed two strategies to prevent relapse after successful treatment: Insight Cards and Value-Based Goal Setting (VBG). Insight Cards consisted of a set of cards on which patients could write down their most meaningful rehabilitation experiences, ideas, and milestones (Multimedia Appendix 1). The upper half of the card provided space for the insights, and the bottom half was reserved for a related environmental cue such as a picture or a quote. The collection of these cards allowed HCPs to ensure the intervention was received as intended (ie, teach-back) and evaluate progress. For patients, the collection of Insight Cards provided lasting access to their most meaningful rehabilitation experiences in their personal environment.

VBG consisted of a worksheet that facilitated the formulation of meaningful goals. The first part of the worksheet prompted patients to identify important personal values. The second part consisted of a prespecified algorithm to formulate desirable and feasible goals that were related to one of the identified values and could be attained within 6 months. Patients were also encouraged to set up calendar reminders and organize social support. In the third part of the worksheet, patients could plan their goal-directed activities in multiple steps, which facilitated gradual progress toward the goal. For each step, patients indicated what, when, and where as they planned the activity. Next, patients were prompted to identify potential barriers and formulate adequate strategies to overcome these barriers. When the first step was completed, patients could plan consecutive steps until the goal was attained. By continuously using the same step-by-step sequence, patients learned to set desirable and feasible goals for themselves and progress toward attainment through achievable steps, while anticipating potential barriers (Multimedia Appendix 2). Multimedia Appendix 3 provides an overview of the prototype intervention components and behavior change techniques (BCTs), according to the BCT taxonomy V1 of Michie and colleagues [26]. The Dutch version of the full workbook is available in Multimedia Appendix 4.

Both strategies were presented in the prototype workbook. This end result of the co-design project was not only based on cocreation and user experience evaluations but also informed by behavior change theory [27,28]. To optimize the fit between the prototype intervention and the needs of each patient, we developed three workbook versions. Two workbook versions contained either VBG or Insight Cards. The third workbook version contained both strategies.

### Procedure

We wanted to minimize the impact of this study on existing treatment procedures to resemble a real-world situation as much as possible. Therefore, we did not prescribe when, how, or how much the workbook should be used. Rather, treatment teams were free to select the appropriate patient and time point for introducing any of the workbook versions. For each participating treatment team, a 1-hour training session was provided in which the content of the workbook and suggestions for integrating the strategies into the treatment program were explained (eg, by encouraging patients to use an Insight Card to express a particular relevant treatment experience). Patient inclusion was permitted at any time during treatment, as long as the HCPs considered the workbook to be of potential additional value. We recommended HCPs discuss the workbook with patients on a weekly basis during treatment, but they were free to decide how and when to use it. For a period of 6 months, printed workbooks were made available to the teams.

When the treatment staff decided to introduce the workbook, patients were approached by their physician or therapist who explained the study purpose and provided an information letter that explained the purpose of the study. Patients who were willing to participate signed an informed consent form and were provided with instructions on how to use the workbook by a member of the treatment staff.

### Data Collection

#### Patient Interviews

Approximately 1 month post-IMPT, participants were contacted for a semistructured telephone survey of approximately 20 minutes by SE (male, physical therapist psychologist and as researcher involved in the development of the prototype intervention). The researcher was not involved in the treatment program, and the telephone survey was the first contact with the participants. In the introduction, the researcher explained the study aim and his role in this project. He also encouraged patients to speak frankly and provide all information that could be relevant for future use or development. Patients were asked to describe (1) the frequency of using the workbook, (2) the effect of the workbook on treatment adherence, (3) their satisfaction with using the workbook, (4) the contribution of the workbook to positive behavior change, and (5) its overall usability. Each topic was introduced with an open question (eg, “To what extent does the workbook facilitate the maintenance of treatment goals at this moment?”) and followed up with one probing question (eg, “Could you give an example of how the workbook facilitated the maintenance of treatment goals?”). After each answer, the interviewer repeated the notes to check if they accurately reflected the meaning of the patient’s responses. In response to our request to participate in an in-depth follow-up interview of 60 to 90 minutes to provide information about using the workbook over a prolonged follow-up time (approximately 10 months posttreatment), two participants agreed. We used a general interview guide approach to ensure that each topic of interest would be covered while adopting a flexible and responsive attitude to the participant feedback [29]. The topic list queried experiences with the workbook during and after treatment, suggestions for improvement with respect to layout and content, and ideas for integrating the workbook in the treatment program. We developed interview guides for both interviews, each containing a topic list with guiding questions and a list of procedures (eg, testing the recorder). The topic lists contained introductory questions to build rapport and make participants comfortable with the topic, key questions that focused on obtaining the information of interest, and ending questions to check if anything of relevance was missed.

#### Focus Group

Four members of the rehabilitation team in Maastricht who were experienced with using the workbook participated in a 90 minute focus group session. This was held 12 weeks after the experimental phase and moderated by two researchers (AK/JP, both male, PhD, physical therapists and as researchers involved in the development of the workbook). Two analysts (SE/SB) were present to take notes and record the session. Similar to the interviews, we developed a focus group guide that included procedures, task assignments, and 9 open-ended questions [30] (Multimedia Appendix 5). During the session, the moderator ensured that all participants had sufficient opportunity to express their thoughts and ask clarifying questions when necessary. Each question concluded with a short summary before the group moved on to the next question.

### Data Analysis

The dataset for the qualitative analysis consisted of verbatim transcripts of the focus groups and patient interviews and the notes that were taken during the telephone surveys. All files were imported into Atlas.ti version 8.4 (Scientific Software Development GmbH) for analysis. We adopted a deductive thematic approach to identify, analyze, organize, describe, and report the themes that we found within our data [31-33]. Importantly, thematic analysis enables researchers to summarize the most important topics of a dataset using a stepwise approach that involves coding all data segments relevant to the research question. We constructed a deductive framework a priori that consisted of 3 themes we believed to be essential for determining the feasibility of the prototype intervention. We considered a theme as a meaningful group of data segments representing a phenomenon of interest in relation to the study question [31,32,34]. Applicability (theme 1) refers to the extent and manner in which the workbook could be integrated in the existing treatment program [35]. Acceptability refers to the extent by which the workbook is evaluated as suitable, satisfying, or attractive [23,36]. We were not only interested in how participants judged the acceptability of the workbook content (theme 2), but also they evaluated the presentation form (theme 3). We added this latter theme because the current presentation form was chosen for practical purposes and we remained interested in alternative ideas.

In the first step of the data analysis, researchers read the data several times. All potentially relevant segments were coded according to these broad themes, but we allowed the possibility of adding extra themes if that would lead to a more accurate insight into the feasibility of this prototype intervention. In the second step, we inductively organized the data segments into subthemes to accurately describe the content [32].

SE and AK independently analyzed the patient data, and SE and JP independently analyzed the HCP focus group transcripts. Each step contained several iterations where researchers discussed the meaning of the data as well as how to accurately describe the data in terms of themes and subthemes with respect to the study aims. At the end of this process, the researchers held a final consensus meeting that involved summarizing the data in themes, subthemes, related quotes, and interim conclusions.

Although the answers on key questions during the interviews and focus groups were summarized by the interviewer to confirm they sufficiently captured their experience or opinion, participants were not involved in checking the results of the data analysis.

## Results

### Demographics

During the course of the study, a workbook was offered to 19 patients; 8 patients did not respond to our requests to participate in the telephone survey. Therefore, our final dataset came from a focus group with 4 HCPs, telephone interviews with 11 patients, and in-depth interviews with 2 of these patients. The HCPs were a behavior therapist (male, 19 years’ experience), physiotherapist (male, 5 years’ experience), behavior therapist (female, 14 years’ experience), and occupational therapist (female, 11 years’ experience). Table 1 provides an overview of the patient participant characteristics.

The majority of codes could be clustered within our predefined framework, but we added the theme adaptation for a better fit. This theme covered the extent to which the prototype intervention could be adapted to each patient or whether personal characteristics were required for effective use. Table 2 provides an overview of our final coding scheme.

**Table 1:** Overview of patient participant characteristics (n=11).

Characteristics	Value
Female, n (%)	6 (55)
Age in years, mean (SD)	55.20 (12.21)
Pretreatment level of disability (PDI^a^), mean (SD)	35.29 (9.48)
Pretreatment level of anxiety (HADS^b^), mean (SD)	8.83 (3.55)
Pretreatment level of depression (HADS), mean (SD)	8.00 (4.52)

^a^PDI: Pain Disability Index.

^b^HADS: Hospital Anxiety and Depression Scale.

**Table 2:** Coding scheme, including themes, subthemes, and representative quotations.

Themes and subthemes		Representative quotations
**1. Applicability of the workbook in the existing treatment programs**		
	1.1 Introduction of the intervention (48 quotations)	I would have preferred the assistance of a therapist with the formulation of my first value-based goal. Without help, it took me some time to understand the logic behind the procedure. [P8^a^]
	1.2 Interaction with the treatment program (35 quotations)	The workbook fits the treatment program well. [P11]
	1.3 Final phase of treatment (7 quotations)	I liked to review the Insight Cards during the follow-up session. One patient even attached photos to his cards. It was nice to browse through and to gain insights in his experiences. [T2^b^]
	1.4 Role of health care provider (28 quotations)	I liked the support of the therapists. During the sessions, we went through the workbook and discussed everything. They also reminded me sometimes to write down experiences. [P3]
**2. Acceptability of the workbook content**		
	2.1 Usage (33 quotations)	I did not actively use the workbook posttreatment, but it is nice to have it as a work of reference. [P17]
	2.2 Action mechanisms (47 quotations)	The workbook was an essential element in the process of learning to understand my condition. [P2]
	2.3 Reported outcomes (30 quotations)	For me, the workbook functioned as an extension of the treatment. I could see the program evolve, and patterns change. I could read back my personal development. [P8]
	2.4 New components (27 quotations)	Specific information on triggers that could cause a relapse is not provided in the current prototype. [T3]
**3. Acceptability of the workbook form**		
	3.1 Type of delivery (mobile health vs paper and pencil; 32 quotations)	A negative point for both is that the intervention content would better fit an eHealth intervention in the current time. [T2]
	3.2 Written instructions (18 quotations)	Related to Value-Based Goal Setting: I believe the concept [of values] is difficult. For example, one patient did not understand the idea behind “source of inspiration.” People that you needed to look up to...she found that a scary idea. [T4]
	3.3 Appearance (13 quotations)	I had difficulty with the initial structure of the layout. How does that work? [P8]
**4. Adaptability**		
	4.1 Personal characteristics (45 quotations)	To use the workbook appropriately, patients will need some sort of self-reflecting skills, as well as intrinsic motivation. [T1]

^a^P: patient.

^b^T: health care provider.

### Applicability of the Workbook in Existing Treatment Programs

Based on their own experiences, patients and HCPs discussed how the workbook could be optimally implemented and adopted throughout the treatment program. Regarding the introduction of the workbook, there was no consensus among HCPs about when to introduce the workbook during treatment. An early introduction of VBG was believed to facilitate the formulation of treatment goals at the start of the program, and Insight Cards were considered useful to immediately capture important experiences at the start of the program (eg, the initial experiment of exposure in vivo). On the other hand, HCPs were hesitant to add more instruction time to an already information-dense start of the program.

If you provide patients with the workbook at the start of the program, you will also have to educate them on how it should be used. ... Yes, this will add to an already long queue of things that require explanation.

T2

From the patients’ perspective, an early introduction did not seem crucial: patients who received the workbook in later stages still evaluated its use as relevant.

Although patients reported substantial variation on time spent on the workbook during treatment sessions ranging from discussing the workbook during each contact to no integration at all, HCPs found Insight Cards easy to integrate into their therapy sessions. The interaction with the treatment program was more difficult for VBG.

Sometimes, value-based goals relate to higher order goals than the goals we can work with. If you start discussing patients’ values, you can fill plenty of sessions. However, this will be a different type of treatment than we are currently providing.

T1

In general, HCPs commented that regular checks of the content were preferable within treatment contact time but that the total time spent should be limited. Both patients and HCPs specifically considered these checks important during the final phase of treatment. For example, a patient commented on whether it would be relevant to evaluate the workbook during the final phase of treatment.

Yes, absolutely. That makes sense to me. It is not necessary to discuss the workbook every week, but it would help to ask at certain moments how things are going. Then, patients can show how they are using the workbook. They might be using it improperly. That sometimes by evaluating and also discuss this during the final evaluation: what’s the status? How far did you come?

P2

Patients reported mixed experiences relating to the role of the health care provider regarding the workbook. Active involvement was considered useful as it facilitated the transition from the workbook to treatment and vice versa. Patients who did not actively discuss the workbook with their HCPs believed that more involvement would have led to better outcomes. As a minimum, they recommended an HCP-led introduction where the use of the workbook would be explained.

### Acceptability of the Workbook Content

Patients and HCPs reflected on what potential action mechanisms were involved and which behavioral outcomes were targeted. Participants reported that the use of Insight Cards helped to create a moment of reflection on important experiences. Also, rereading the experiences provided a better understanding of important treatment concepts. For VBG, patients indicated that it contributed to pacing of activities, planning meaningful goals, and anticipating the effort needed to attain the goals. One patient who used the VBG strategy to plan a long journey in advance commented.

A positive experience is ... Normally it is just persisting, no matter what. I will do this now. But here, if you aim for greater things, you will need to work towards it. I have seen that clearly now.

P8

Patients reported a shift from active use during treatment to passive use (as a work of reference) posttreatment.

I have not actively used the workbook after treatment, but I am glad that it is available as a reference book.

P17

During active use, VBG especially was considered time-consuming, which hindered regular use for some patients. In the posttreatment phase, one patient reported that reading the workbook content helped him counter an impending relapse. Participants made multiple suggestions for new components to the prototype intervention such as additional reading material on pain education, a specific section that describes the possibility of relapse, and the option of prioritizing the most important Insight Cards. Participants further suggested discussing the workbook with peers during group meetings and made specific suggestions should the workbook be developed into a digital app, such as adding informative video clips and a digital avatar that could interact with the patient.

Participants reported that the workbook contributed to facilitating the pursuit of meaningful goals, providing a structure to the treatment process, pacing activities, monitoring progress, and revealing the most important milestones during the program.

A patient started with the Insight Cards at the final treatment phase. Nevertheless, during the refresher day his workbook was an exemplar of how they [the Insight Cards] should be used. Completely filled out and illustrated with photographs. He also mentioned that, in case of potential relapse, he could imagine himself using the workbook and browsing through his experiences.

T2

These positive outcomes were not shared by all participants. Some patients reported that the intention of the prototype intervention was not clear or questioned its efficacy.

I wonder whether a workbook would be sufficient to ensure the transfer to the home situation.

P13

Also, HCPs were cautious that too much emphasis on personal values could cause patients to focus on topics and goals that were beyond the scope of the treatment.

### Acceptability of the Workbook Form

The general appearance and structure was appealing to most participants, but some patients provided suggestions for reordering the workbook and moving all instructions to the beginning. For VBG, the written instructions were considered too elaborate and complex, which caused confusion and problems in understanding.

It [the workbook instructions] should be easier to read. It may be due to my short attention span or because I am not a good reader, but I did like the underlying idea.

P9

The instruction text for the Insight Cards was shorter and easier to understand. Participants indicated that the included examples were helpful for both relapse prevention strategies.

Overall, participants would prefer a mobile health (mHealth) app over a printed workbook as type of delivery. In particular, the possibility of combining the strategies with smartphone functionalities such as a calendar and camera could lead to more personalized experiences and goals. Moreover, a digital app would be accessible throughout the day, allowing patients to directly record experiences, browse through insights, or plan new goals.

I would prefer an app. An app can provide feedback and is able to alert you. For example, I will plan a 3k walk for tomorrow. Then I will receive a reminder that I should go for a 3k walk. If think “no! not tomorrow,” then—it is quite simple—I will modify the calendar...this is the future.... I have not grown up with this thing [mobile phone]. But nowadays people only look on their phones throughout the day. They will benefit more from this [the app] than from this paper [the workbook]. So I would think that is very important.

P8

### Adaptability

The theme adaptability was added in response to multiple comments relating to individual personal characteristics that were believed to either facilitate or hinder optimal use of the workbook. In particular, participants reported that the current version of the prototype intervention required a high level of commitment and an active mindset to autonomously explore the features of the workbook.

Because of my work, I am used to discover things on my own, but I expect that this method will not work for everyone if it is not clearly instructed.

P8

Patients with low health literacy were expected to encounter problems with the current amount of instruction texts, reading level, writing down their own input, and analyzing which values would underlie their most important treatment goals. Characteristics that were reported to facilitate the use of the workbook included being organized, being able to reflect on experiences, and possessing adequate reading and writing skills.

## Discussion

### Principal Findings

This study was conducted to assess the feasibility of two relapse prevention strategies specifically designed to enhance IMPT programs. Overall, the workbook was perceived by participants as useful, easy to use, and in line with the treatment program. However, there was a difference in how the individual relapse prevention strategies were perceived. Insight Cards were expected to benefit all patients and were relatively easy to learn and apply. VBG helped patients to plan meaningful goals, but these were more difficult to understand and did not always fit into the treatment program. However, it is important to note that the focus group consisted solely of HCPs who provided exposure in vivo treatment and were not experienced with VBG. Participants indicated a preference for a digital app over a paper-and-pencil workbook as a future delivery mode. Other suggestions for improvement included more specific implementation guidelines for the treatment staff, group sessions among patients to discuss their input, and more attention to the workbook during the final phase of the treatment. Overall, these findings indicate that the workbook is feasible within the context of IMPT and acceptable to both patients and HCPs.

Importantly, these initial results contain detailed feedback on how the strategies can be refined. First, the study protocol allowed for substantial variation in when and how the workbook was applied. This flexible approach maximized HCP autonomy with respect to dose and content but at times resulted in limited or even no interaction at all once the workbook was introduced. As a consequence, not all intervention components were used effectively by all participating patients. For example, HCPs could use the Insight Cards to check if the patient understood important treatment principles as intended, but the dataset includes no mention of such a teach-back occurrence. Therefore, we believe that, in line with other study findings, an extensive onboarding procedure with additional guidelines, examples, and training sessions would improve overall implementation and optimize the potential of the workbook [37-39]. Based on the evaluations in this study regarding patient characteristics and requirements for optimal use, this onboarding procedure could also contain a deliberate consideration whether either or both interventions may benefit a patient. Second, the VBG sequence needs revision to improve clarity for patients and ensure that the goal-setting procedure matches the treatment program. The sequence was based on the insight that value-based goal-setting procedures outperformed specific, measurable, achievable, relevant and time bound (ie, SMART) goal setting [40,41]. However, patients reported difficulties in understanding the concept of values through written instructions in the workbook, particularly when the treatment program did not structurally include a values assessment. Altering the VBG procedure to shift the initial emphasis from values to goals may increase clarity; patients could begin formulating specific goals related to improved physical functioning instead of starting with personal values. Subsequently, assessing why this particular goal is relevant to the patient could direct attention toward associated values. Third, HCP responses concerning the amount of time spent with the prototype intervention suggests that they experienced a trade-off between focusing on the treatment program or preventing relapse with the workbook. This indicates that relapse prevention with the current version of the workbook is not yet perceived as an integral aspect of the treatment. Given that the workbook was introduced as an addition to the existing IMPT, this finding is not surprising. Nevertheless, future development should take time efficiency into account and focus on increased integration of the relapse prevention strategies into the existing treatment protocols. One possibility is to relate the identification of problems in daily life functioning during the assessment phase (eg, by using instruments such as the Canadian Occupational Performance Measure) to the goal-setting procedure of VBG. Furthermore, integration of Insight Cards into clinical practice could be enhanced by routinely relating this to specific communication strategies, such as a teach-back approach [42,43]. Reflecting on Insight Cards during patient-therapist conversations could facilitate both shared decision making and teach-back and empower patients to actively participate.

One promising direction for the development of the prototype is to embed these strategies in an mHealth app (ie, software apps designed for mobile devices to provide or support health care services) [44]. This domain is becoming increasingly important in the assessment and treatment of chronic pain and is particularly suited for tailoring specific strategies to individual needs and preferences [45]. With machine learning approaches, it is even possible to automate the process of personalizing the strategies based on user-generated data [46]. Another advantage of mHealth is the opportunity of letting both strategies interact. For example, if a patient used an Insight Card to highlight an effective strategy to overcome barriers to physical activity, this card could also be used as a future solution to anticipated problems within the planning procedure of VBG. Although the idea of a digital intervention had already been suggested by stakeholders in earlier development stages, we did not make any decisions on its final form prior to this feasibility study. Because our study findings are in accordance with these earlier suggestions, we believe there is potential in transferring this prototype workbook into an mHealth app.

### Limitations

Limitations of this study include a small sample size and relatively high dropout rate. Because the study was designed, conducted, and analyzed by the same three researchers, who were also involved in the development of the workbook, confirmation bias and socially desirable responses may have resulted. Furthermore, due to organizational reasons at location Hoensbroek directly after the inclusion period, we were only able to collect evaluations from HCPs from location Maastricht, where regular reflections on patients’ values are beyond the scope of their treatment program. With these limitations in mind, it is important to reflect on the validity of the conclusions of this study. Concerning the adequacy of the sample, Malterud and colleagues [47] have introduced the concept of information power, which is determined by five factors: narrow or broad study aim, sample specificity, established underlying theory, quality of dialogue, and type of analysis strategy. We believe that the specific focus in our study objective on 3 key factors for feasibility positively contributed to the information power of this dataset. Furthermore, all participants that we interviewed received instructions to use the workbook, participated in an IMPT program, and had—at minimum—made an effort to use the workbook in this setting, which not only resulted in high specificity but also to a high quality of dialogue. In addition, we included several established procedures to enhance the credibility of our findings and minimize bias, including member checks, triangulation of researchers and data sources, and including questions regarding negative experiences with the workbook to search for disconfirming evidence [32,48,49]. However, we conducted a cross-case analysis and the low sample size resulted in limited variation on personal characteristics and a low likelihood that potential problems in use did occur within the sample [47,50], which limits the generalizability of our findings.

Although multiple reasons could have contributed to these limitations, an important factor may have been our real-world approach toward the use of the prototype intervention within the inclusion period. We expected that the active participation of the treatment teams during previous development phases would contribute to high patient inclusion rates. However, it is likely that the limited guidance on when or how to explain the workbook and absence of fixed procedures regarding patient recruitment increased the required effort for HCPs to integrate this study into their treatment routine. Although this means that the extent to which this workbook can work in IMPT programs is inconclusive, we did obtain important insights for further developing the prototype intervention. From an intervention design perspective, the feedback from actual use within the intended environment is crucial to further refine the strategies and adapt them to that specific context [51]. Regarding the low response rate on the in-depth interviews, some patients indicated that they already provided a full evaluation of the workbook in the telephone interview. Other patients mentioned the traveling distance as main reason. In addition, we believe that a moment of direct contact with the researchers prior to the telephone interview could have helped to better explain the importance of the interview and establish a good rapport in advance. For these reasons, this study should be regarded as the first iteration in the overall process of transforming a prototype into an effective intervention for clinical practice. Czajkowski et al [52] emphasize the need for initial prototyping before conducting more stringent tests in order to first align the behavioral strategies to the clinical context in which they will be implemented. In addition, experimental medicine highlights the need for a stepwise approach toward intervention development. This framework consists of multiple subsequent steps that should be undertaken to examine the relationships between the intervention and its effect on physical functioning and the modifiable behavioral factors that mediate this relationship [53]. Consequently, further development and testing are required and should indicate whether these strategies lead to a change in specific health behaviors such as goal setting and problem-solving and to what extent this change causes clinically relevant long-term improvements for patients with chronic pain. In addition, these limitations provide valuable information in preparation of future trials, including more emphasis on training HCPs in how to use the strategies, more integration of study procedures within clinical practice, and improved patient fidelity procedures to decrease dropout.

### Conclusion

This first test of the relapse prevention workbook in a real-world setting of IMPT programs resulted in important insights regarding form, content, and use, as well as its interaction with the treatment program and study design. Although these initial results indicate a favorable evaluation of behavior regulation strategies within the workbook, this study encountered multiple barriers regarding implementation and data collection that limit the generalizability of these results. Future studies should address the fidelity of HCPs and patients and should include clear procedures regarding recruitment and use of both relapse prevention strategies during treatment. Future development efforts should consider eHealth or mHealth options, extensive onboarding, and a modified value-based goal-setting procedure for the VBG strategy.

## Appendix

Multimedia Appendix 1 Insight Card examples with a blank card (translated from workbook).

Multimedia Appendix 2 Value-Based Goal-Setting blank form with example (translated from workbook).

Multimedia Appendix 3 Overview of intervention components of Value-Based Goal Setting and Insight Cards with corresponding behavior change techniques.

Multimedia Appendix 4 SOLACE workbook in Dutch.

Multimedia Appendix 5 Focus group guide with procedures, task assignments, and questions (translated from workbook).

## Abbreviations

BCT: behavior change technique
HCP: health care provider
IMPT: Interdisciplinary multimodal pain therapy
mHealth: mobile health
SMART: specific, measurable, achievable, relevant and time bound
VBG: Value-Based Goal Setting
