# Supplementary material for: Exploring the Feasibility of Relapse Prevention Strategies in Interdisciplinary Multimodal Pain Therapy Programs: Qualitative Study
Source: JMIR Hum Factors. 2020 Dec 11;7(4):e21545. doi: 10.2196/21545 (PMC7762683; doi:10.2196/21545)
Supplement: Multimedia Appendix 1 [file humanfactors_v7i4e21545_app1.pdf]

SUPPLEMENT 1

TRANSLATED EXCERPT FROM WORKBOOK: TWO EXAMPLES AND AN EMPTY INSIGHT CARD.

MOMENT IT HELPS TO TALK ABOUT THE TREATMENT PROGRAM:
MY WIFE IS NOW LESS WORRIED WHEN I GO FOR A WALK.

Memory Cue

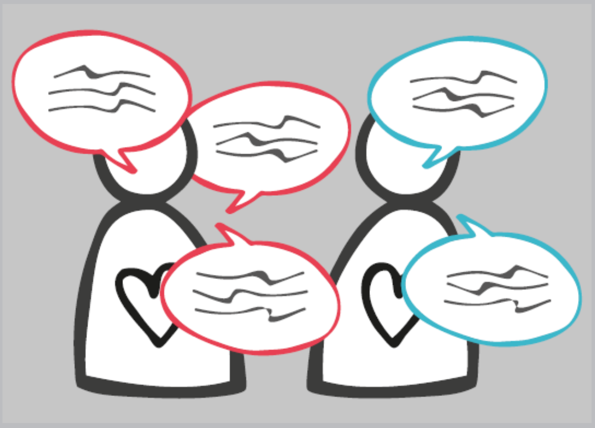

MOMENT IF I AM IN PAIN, IT DOES NOT HAVE TO MEAN THAT
SOMETHING IS BROKEN OR INJURED.

Memory Cue

HURT ≠ HARM

MOMENT

MEMORY CUE
