# Supplementary material for: Exploring the Feasibility of Relapse Prevention Strategies in Interdisciplinary Multimodal Pain Therapy Programs: Qualitative Study
Source: JMIR Hum Factors. 2020 Dec 11;7(4):e21545. doi: 10.2196/21545 (PMC7762683; doi:10.2196/21545)
Supplement: Multimedia Appendix 2 [file humanfactors_v7i4e21545_app2.pdf]

SUPPLEMENT 2

TRANSLATED EXCERPT FROM WORKBOOK: VALUE BASED GOAL-SETTING FORM, INCLUDING AN EXAMPLE.

MY VALUE

MY GOAL

Check 1: How desirable is this goal for you?

The chances of successfully completing a goal are lower, when the goal is not important to you.

not at all desirable *minimum* *very desirable*

Check 2: How confident are you that you can succesfully achieve this goal?

If you are confident in your own capabilities, you are more likely to attain this goal.

Not confident at all *minimum* *very confident*

Check 3: How much time will it take to achieve this goal

one day *6 months*

If you estimate that goal achievement will take more than 6 months, we recommend to divide this goal into multiple subgoals. You can describe the first subgoal below:

Check 4: Is there someone who you can ask for help?

Check 5: Add a calender reminder?

YES NO

REWARD

THE FIRST STEP

What will I do?

Where will I do this

WHEN WILL I DO THIS?

- Today
- Tomorrow
- day after
- ELSE:

OBSTACLE

ACTION

NEXT STEP

What will I do?

Where will I do this?

WHEN WILL I DO THIS?

- today
- tomorrow
- day after tomorrow
- ELSE:

OBSTACLE

ACTION

NEXT STEP

What will I do?

Where will I do this?

WANNEER GA IK DIT DOEN?

- today
- tomorrow
- day after tomorrow
- ELSE:

OBSTACLE

ACTION

GOAL ATTAINED?

- YES, *see page xx.*
- No, I need more steps.
See page xx for more steps
- No, this goal was not the right choice.
see page xx.

MY VALUE

ACTIVITY

MY GOAL

IN FOUR WEEKS, I WANT TO JOIN A 5K WALK DURING A LOCAL WALKING EVENT.

Check 1: How important is this goal for you?

The chances of successfully completing a goal are lower, when the goal is not important to you.

Not important at all — *minimum* — *Very important* (marked: X near Very important)

Check 2: How confident are you that you successfully attain this goal?

If you are confident in your own capabilities, you are more likely to attain this goal.

Not confident at all — *minimum* — *Very confident* (marked: X just above minimum)

Check 3: How much time will it take to reach this goal?

One day — *6 months* (marked: X)

If you estimate that goal achievement will take more than 6 months, we recommend to divide this goal into multiple subgoals. You can describe the first subgoal below:

Check 4: Is there someone who you can ask for help?

MY HUSBAND WILL JOIN ME

Check 5: Add a calender reminder?

X YES ○ NO

REWARD

WE WILL CELEBRATE THIS WITH A NIGHT OUT

THE FIRST STEP

what will I do? I CHALLENGE MYSELF TO GO FOR A 5K WALK

where will I do this? IN THE FOREST NEARBY

WHEN WILL I DO THIS?

- [x] Today
- [ ] Tomorrow
- [ ]
- [ ] ELSE:

OBSTACLE A HEAVY DOWNPOUR

ACTION I IMMEDIATELY PLAN A NEW MOMENT LATER DURING THE WEEK.
