# Supplementary material for: Exploring the Feasibility of Relapse Prevention Strategies in Interdisciplinary Multimodal Pain Therapy Programs: Qualitative Study
Source: JMIR Hum Factors. 2020 Dec 11;7(4):e21545. doi: 10.2196/21545 (PMC7762683; doi:10.2196/21545)
Supplement: Multimedia Appendix 3 [file humanfactors_v7i4e21545_app3.pdf]

SUPPLEMENT 3

Overview of intervention components of both strategies with corresponding Behavior Change Techniques.

Table S3. Overview of intervention components of both strategies with corresponding Behavior Change Technique.

Prototype 1: Value-Based Goals

	Component	Behavior Change Technique
**A.**	**Select personal values**	
	Instruction to identify important personal values, including various optional generative techniques to identify personal values (e.g. a list of frequently used values).	13.4 Valued self-identity
**B.**	**Formulate specific value-based goals**	1.1 Goal setting (behavior)
	Instruction to formulate a feasible and desirable goal that relates to a personal value and can be attained within six months. The worksheet includes prompts to seek social support, to set calendar reminders and to choose a reward when the goal is attained.	1.3 Goal setting (outcome)<br>3.1 Social support (unspecified)<br>7.1 Prompts/cues<br>10.7 Self-incentive
**C.**	**Formulate a first step**	
	Instruction to formulate the first activity towards goal attainment, including specification of the where, when and how. Includes anticipating potential obstacles and planning coping strategies.	1.2 Problem solving<br>1.4 Action planning
**D.**	**Formulate subsequent steps**	
	Subsequent steps towards goal attainment can be planned using the same procedure. This worksheet also includes instructions to reflect on the process if the goal is attained or abandoned.	1.2 Problem solving<br>1.4 Action planning<br>1.7 Review outcome goal(s)
**Prototype 2: Insight Cards**		
**A.**	**Describe a meaningful experience**	
	Instruction to create a collection of Insight Cards, by writing down the most relevant experiences at regular moments during the treatment program. The upper half of each card contains space to write down meaningful experiences, ideas and milestones. The bottom half is reserved for	2.3 Self-monitoring of behavior<br>7.1 Prompts/cues<br>7.8 Associative learning<br>11.3 Conserving mental resources

associated quotes, pictures or other types of related cues.	
**B. Teach-back essential treatment components**	
Instructions for HCPs to discuss the Insight Cards to check if key elements in the treatment program were received and understood as intended.	4.1 Instruction on how to perform the behavior<br>13.2 Framing/reframing
**C. Create a patient-specific overview**	
Instructions for HCPs at the final treatment phase to review the Insight Card collection to assess progress, review the program, or to focus on specific key moments.	15.3 Focus on past success
**D. Use personal experiences as reference work**	
Suggestion for patients to review their most important experiences and insights of the program during the post-treatment phase in general, but specifically in case of an impending relapse. The Insight Cards were delivered with a refrigerator magnet and a card holder that facilitated showcasing specific insights in the associated environmental context.	5.2 Salience of consequences<br>12.5 Adding objects to the environment
