# Supplementary material for: Exploring the Feasibility of Relapse Prevention Strategies in Interdisciplinary Multimodal Pain Therapy Programs: Qualitative Study
Source: JMIR Hum Factors. 2020 Dec 11;7(4):e21545. doi: 10.2196/21545 (PMC7762683; doi:10.2196/21545)

WERKBOEK PIJNREVALIDATIE

HULPMIDDELEN OM UW ERVARINGEN UIT DE REVALIDATIE TOE TE PASSEN IN UW DAGELIJKS LEVEN

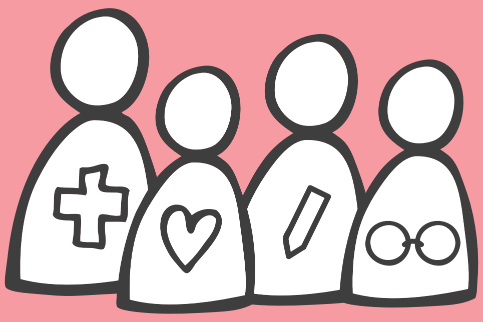

SOLACE

SELF MANAGEMENT IN CHRONIC PAIN STRATEGIES

Projectleider SOLACE

Dr. Jan Pool [1]

Contactpersoon Adelante

Dr. Albére Köke [2,3]

a.koke@adelante-zorggroep.nl

Contactpersoon Hogeschool Utrecht

Stefan Elbers [1, 3]

stefan.elbers@hu.nl

Vormgeving

Rianne Schaekens

www.solace.hu.nl

[1] Lectoraat leefstijl en gezondheid, Hogeschool Utrecht

[2] Kenniscentrum, Adelante Zorggroep, Hoensbroek

[3] Onderzoeksinstituut CAPHRI, Universiteit Maastricht

adelante haal het beste uit jezelf

Maastricht University

HOGESCHOOL UTRECHT

Lectoraat leefstijl en gezondheid
Lectoraat crossmediale communicatie in het publieke domein
Lectoraat co-design.

SAMENWERKINGSVERBAND Pijnpatiënten naar één stem

de Fysiotherapeut Koninklijk Nederlands Genootschap voor Fysiotherapie

SiA Nationaal Regieorgaan Praktijkgericht Onderzoek

WERKBOEK PIJNREVALIDATIE

HULPMIDDELEN OM UW ERVARINGEN
UIT DE REVALIDATIE TOE TE PASSEN
IN UW DAGELIJKS LEVEN

INTRODUCTIE

Pijnrevalidatie helpt u om een **betekenisvol leven** te leiden met de pijn. Met dit werkboek heeft u ook **na** de revalidatie een hulpmiddel dat u steun kan geven wanneer dat nodig is.

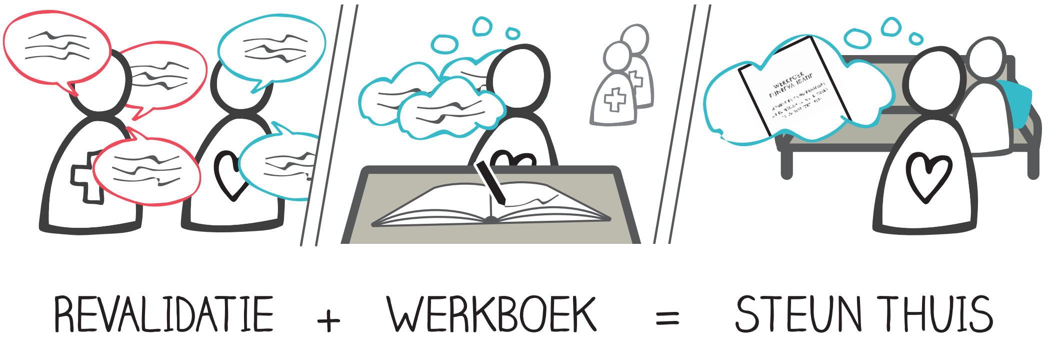

Na de revalidatie is het niet altijd even gemakkelijk om zonder hulp van het team door te gaan. Daarom bieden we u alvast dit werkboek aan, met daarin een hulpmiddel voor later. Door hier al mee te starten tijdens de revalidatie kunt u dit straks na de revalidatie nog beter toepassen.

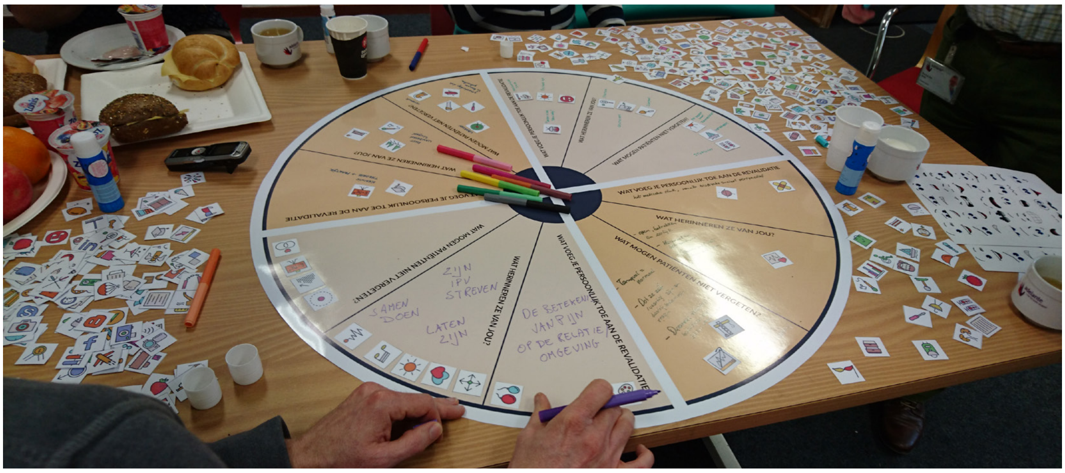

Afbeelding: Patiënten helpen mee met het bedenken van de oefeningen tijdens een ontwerpmiddag.

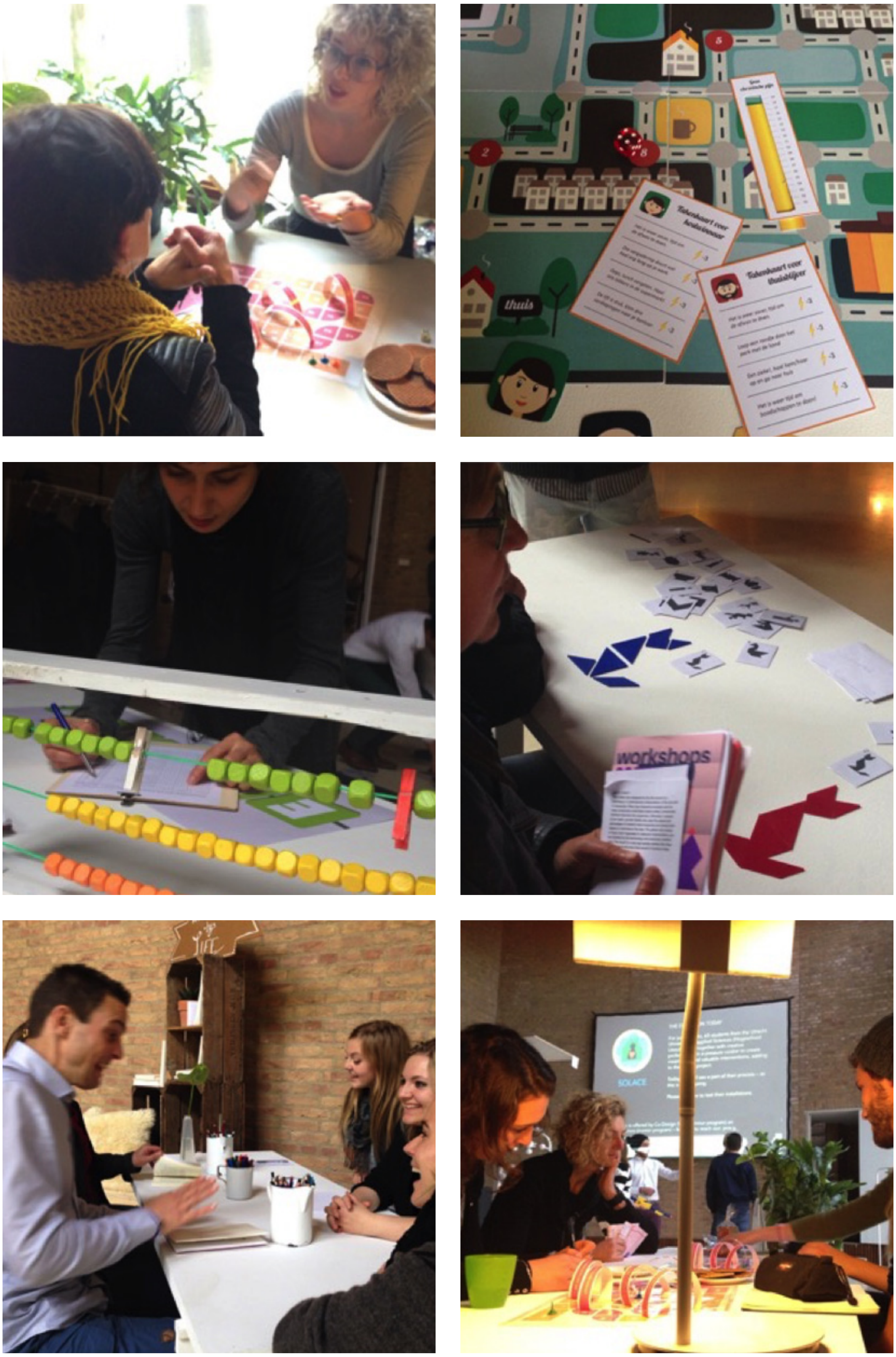

Afbeelding: Studenten die hebben meegeholpen aan dit werkboek presenteren hun ideeën op de Dutch Design Week.

HOE DIT WERKBOEK IS ONTSTAAN: SOLACE

Een revalidatieprogramma helpt u om een **blijvende verandering** in uw dagelijks functioneren en omgaan met de pijn te realiseren. Of deze verandering blijft bestaan als het revalidatieprogramma is afgelopen, hangt mede af van of het u lukt de geleerde vaardigheden blijvend toe te passen in uw persoonlijke situatie.

Om u hierbij zo goed mogelijk te helpen hebben we de krachten van patiënten die het revalidatieprogramma al hebben doorlopen, behandelaren en ontwerpers en onderzoekers gebundeld. Gezamenlijk hebben we dit werkboek ontwikkeld. Hiermee heeft u een hulpmiddel om alles wat u tijdens de revalidatie hebt bereikt vast te houden en eventueel nog verder uit te bouwen.

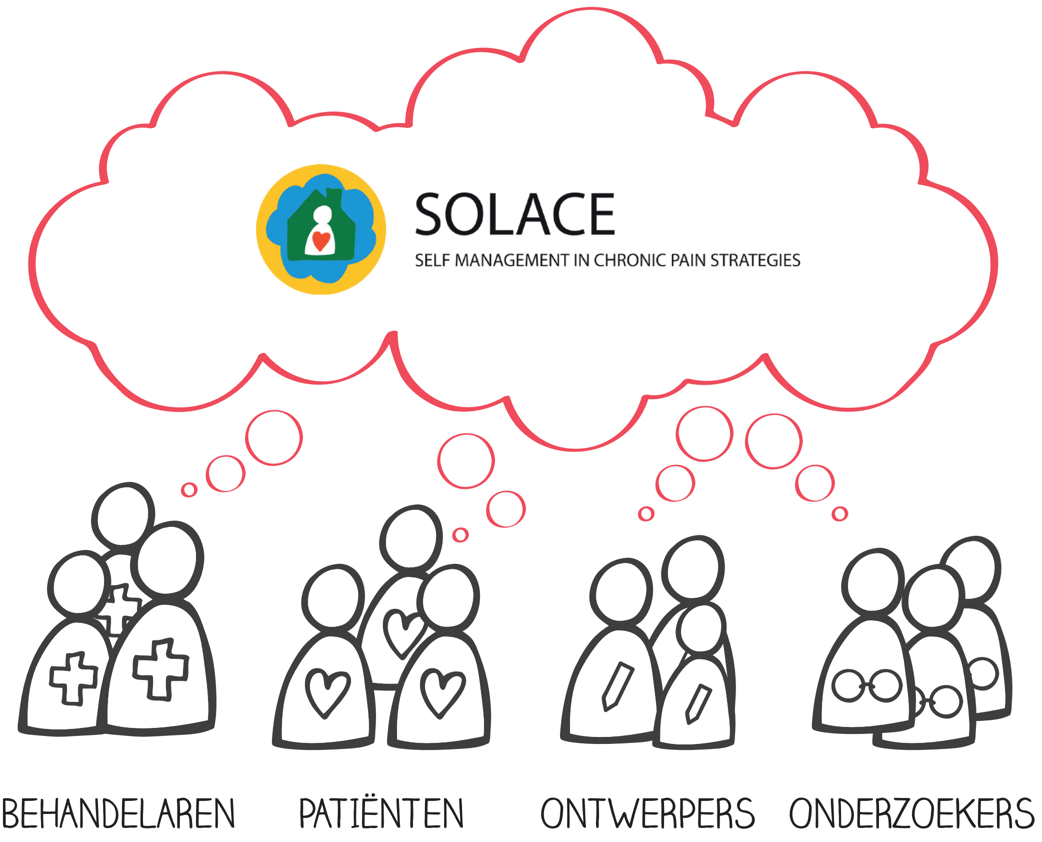

INHOUD VAN DIT WERKBOEK

Na de revalidatie mag u in principe alle activiteiten weer oppakken. Dit werkboek bevat twee oefeningen die u helpen bij het opbouwen en nastreven van activiteiten die waardevol voor u zijn.

PAGINA 11: WAARDEGERICHTE DOELEN

De eerste oefening helpt u om de doelen te vinden die u echt belangrijk vindt in uw leven. Vervolgens kunt u met behulp van een schema stap voor stap toewerken naar het bereiken van deze doelen.

PAGINA 65: DO IT YOURSELF

De tweede oefening helpt u bij het opslaan en bewaren van momenten die voor u belangrijk zijn tijdens de pijnrevalidatie. Dit overzicht kunt u vervolgens gebruiken als persoonlijk naslagwerk en het kan een hulpmiddel zijn bij gesprekken.

De oefeningen zijn slechts bedoeld als hulpmiddel. U kunt die onderdelen uitkiezen waarvan u verwacht dat ze u in de toekomst zullen helpen bij het leiden van een betekenisvol leven.

Als u vragen heeft over de oefeningen kunt u hier altijd met uw behandelaar over spreken. U kunt dan samen bekijken hoe u dit werkboek het beste kunt gebruiken.

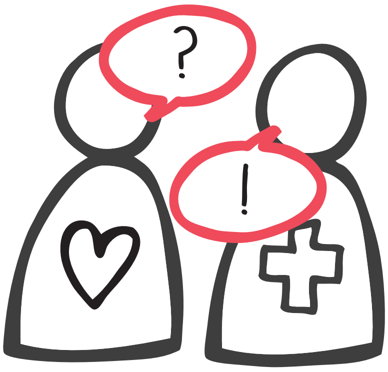

WAARDEGERICHTE DOELEN

Een regelmatig terugkerend doel bij revalidatie is: weer zelfstandig een stuk te kunnen lopen. Maar *waarom* is dit eigenlijk belangrijk? Voor de één betekent dit dat zij haar hond weer zelf kan uitlaten. Een ander kan hierdoor weer boodschappen doen, of naar het café in de buurt.

Een doel in de revalidatie krijgt pas echt betekenis als het een bijdrage levert aan uw geluk of welzijn. Dit noemen we waardegerichte doelen.

De oefening "**waardegerichte doelen**" helpt u uw doelen scherp te krijgen. U gaat in eerste instantie op zoek naar wat belangrijk voor u is. Dit noemen we waarden: persoonlijke idealen die richting geven aan uw handelen. Vervolgens kiest u doelen die aansluiten bij deze waarden en werkt u stapsgewijs toe naar het behalen hiervan. Hierbij kunt u zelf kiezen hoe groot u de stap wilt maken, van kleine tussenstappen naar meteen in het diepe. In het schema "waardegerichte doelen" kunt u alles bijhouden en wordt u geholpen om alvast na te denken over de uitvoering van uw doelen.

KORTE HANDLEIDING
De volgende instructie helpt u bij het invullen van het schema.

1. In **deel A** wordt u gevraagd om na te denken over waarden die voor u belangrijk zijn.
2. In **deel B** gaat u het schema invullen.
3. Op pagina's 32 - 51 vindt u lege schema's die u kunt gebruiken bij het stellen van doelen.

NU IK WEER ZELFSTANDIG EEN HALF UUR KAN LOPEN, KAN IK EINDELIJK WEER GOED ZORGEN VOOR MIJN TWEE HONDEN

~ PATIËNT, 2016

DEEL A: WAARDEN KIEZEN

Een waarde geeft richting aan uw handelen. Waarden zijn zaken waar u enthousiast van wordt.

Wat is uw waarde?

Stel uzelf de volgende vragen:

- In welke activiteit zou u al uw energie in willen stoppen?
  Waarom is dit zo belangrijk voor u?
- Welke prestatie of gebeurtenis zou u met trots of dankbaarheid vervullen?
  Waarom maakt dit u trots?

De antwoorden op de tweede vraag wijzen waarschijnlijk op waarden die voor u belangrijk zijn.

VOORBEELDEN VAN WAARDEN ZIJN:

- Ik wil een goede ouder zijn
- Ik wil sportief kunnen zijn
- Ik wil zelfstandig kunnen zijn

We gaan u hieronder op drie verschillende manieren helpen om uw waarden te vinden. U kunt zelf kiezen welke van deze manieren het beste bij u werkt.

MOMENT VAN GROTE BETEKENIS

De volgende vragen helpen u uw waarden te vinden.

1. Moment van grote betekenis

Denkt u eens terug aan de afgelopen periode. Wanneer had u het gevoel dat u iets waardevols of belangrijks deed?

- Misschien heeft u iemand in uw omgeving kunnen helpen?
- Mogelijk heeft u een succes geboekt of een mijlpaal bereikt?
- Wellicht heeft u thuis, op uw werk, met uw sport of voor uw omgeving iets gedaan waar u een voldaan gevoel van hebt gekregen?

Het maakt niet uit hoe lang dit geleden is, zolang het maar van betekenis voor **u** is geweest. Neemt u ongeveer 5 minuten de tijd om een aantal momenten te bedenken waarbij u het gevoel heeft dat u iets belangrijks of waardevols heeft bijgedragen.

Notitieruimte:

2. De waarde

Als het goed is heeft u een positieve invloed gehad op deze gebeurtenis. Waarom was dit zo belangrijk voor u? Uw waarde vindt u in het antwoord op die vraag.

U kunt de momenten van grote betekenis en de bijbehorende waarden noteren op de volgende pagina. Hieronder staan twee voorbeelden.

VOORBEELDEN

HET MOMENT

IK BEN AFGELOPEN JAAR REGELMATIG OP ZIEKENBEZOEK

GEWEEST BIJ MIJN TANTE.

DE WAARDE

IK VIND LOYALITEIT BELANGRIJK. IK KON OP DEZE MANIER

LATEN ZIEN HOE JE MET ELKAAR HOORT OM TE GAAN.

HET MOMENT

IK BEN GESLAAGD VOOR DE CURSUS

"LEIDERSCHAP & COACHEN"

DE WAARDE

IK VIND HET BELANGRIJK OM ME TE

BLIJVEN ONTWIKKELEN

Op deze pagina kunt u momenten opschrijven, met bijbehorende waarden.

HET MOMENT

DE WAARDE

HET MOMENT

DE WAARDE

HET MOMENT

DE WAARDE

INSPIRATIEBRON

De tweede manier om uw waarden te vinden is: nadenken over een inspiratiebron. Neemt u een moment om na te denken over een persoon die belangrijk voor u is, of die u bewondert. Dit kan een familielid zijn, een beroemd persoon, maar bijvoorbeeld ook een collega. Wat er is zo inspirerend aan deze persoon?

Grote kans dat het antwoord op deze vraag ook een waarde bevat die u belangrijk vindt om na te streven. U kunt de notitieruimte hieronder gebruiken om personen en bijbehorende waarden op te schrijven.

Notitieruimte: MIJN BROER IS ERG BELANGRIJK VOOR MIJ. HIJ STAAT ALTIJD VOOR MIJ KLAAR, ALS IK HULP NODIG HEB. IK BEWONDER ZIJN BEHULPZAAMHEID.

INSPIRATIELIJST

De derde manier om uw waarden te vinden is met behulp van een lijst met waarden. Onderstreep de drie waarden uit de onderstaande lijst die voor u belangrijke drijfveren zijn.

INTELLIGENTIE VEILIGHEID GROEI

SUCCES ZELFRESPECT SAAMHORIGHEID

BEHULPZAAMHEID GENIETEN RESPECT

SPIRITUALITEIT RECHTVAARDIGHEID DELEN

ER ZIJN VOOR ELKAAR ORDELIJKHEID

VERTROUWEN GENEGENHEID RIJKDOM

VERANTWOORDELIJKHEID VERBINDING

EERLIJKHEID VRIJHEID VAARDIGHEID

LUISTEREN CREATIVITEIT EERLIJKHEID

RUIMDENKENDHEID LIEFDE

Beschrijft u vervolgens wat deze woorden voor u betekenen:

Notitieruimte: RESPECT; IK VIND HET BELANGRIJK OM RESPECTVOL BEHANDELD TE WORDEN EN MET ANDERE MENSEN RESPECTVOL OM TE GAAN. DIT GAAT VOOR MIJ OM BELEEFDHEID EN OM EEN POSITIEVE, VRIENDELIJKE EN OPEN HOUDING NAAR ANDEREN TOE.

UW WAARDELIJST

Met behulp van de vorige pagina's heeft u een aantal persoonlijke waarden kunnen formuleren. Noteert u hieronder alle waarden die u hierbij heeft gevonden. Deze waarden worden gebruikt in het waardegerichte doelen schema, dat op de volgende pagina wordt uitgelegd.

MIJN WAARDEN

DEEL B: SCHEMA INVULLEN

Met behulp van de instructie op de volgende pagina's kunt u het waardegerichte doelen schema gaan invullen. Pak een leeg schema ( ) uit de klapper en leg deze voor u neer.

Kies nu de waarde waarbij u een doel wilt stellen en vul deze in op het schema.

DOELEN OPSTELLEN

Met behulp van het stellen van doelen is het mogelijk om een stap te zetten in de richting van uw waarden. Doelen die daarbij passen zijn realistisch, maar tegelijkertijd ook uitdagend en stimulerend. Van een goed doel wordt u enthousiast.

TWEE VOORBEELDEN VAN DOELEN BIJ EEN GEKOZEN WAARDE:

DE WAARDE

ACTIEF ZIJN

HET DOEL

OVER 4 MAANDEN LOOP IK MEE IN DE AVONDVIERDAAGSE (5KM)

DE WAARDE

VRIJHEID

HET DOEL

IK WIL OVER DRIE WEKEN EEN ACTIEVE REIS DOOR

THAILAND MAKEN.

Vul nu uw doel in op het schema.

Het is van belang om een doel op te stellen dat voldoet aan de volgende drie eigenschappen:

1. Het doel is belangrijk voor u
2. U heeft vertrouwen in uzelf om dit doel succesvol uit te voeren
3. U kunt het doel binnen afzienbare tijd behalen

Het volgende onderdeel van het schema controleert of het doel voldoet aan deze eigenschappen.

Bij **check 1** staat een ondergrens weergegeven. Als het doel minder belangrijk voor u is dan die grens, dan adviseren wij u om een ander doel op te stellen dat u belangrijker vindt om na te streven.

Bij **check 2** staat eveneens een ondergrens weergegeven. Heeft u niet voldoende vertrouwen in uw vermogen om het doel te bereiken, dan adviseren wij u ook om een ander doel te kiezen.

Verder kan het handig zijn om alvast na te denken over personen die u kunnen helpen bij het bereiken van dit doel (**check 4**) of over het plaatsen van een herinnering in uw agenda (**check 5**).

Op de volgende pagina vindt u een voorbeeld.

Vul nu de checklist in op uw schema.

VOORBEELD CHECKLIST:

Check 1: Hoe graag wil ik dit doel behalen?

Als het doel niet zo belangrijk voor u is, dan is de kans dat het gaat lukken vaak een stuk kleiner.

Helemaal niet belangrijk — *ondergrens* — X — *Erg belangrijk*

Check 2: Hoe veel vertrouwen heb ik erin dat ik het kan?

Als u er veel vertrouwen in heeft, is de kans dat het gaat lukken vaak een stuk groter.

Helemaal geen vertrouwen — *ondergrens* — X — *Erg veel vertrouwen*

Check 3: Binnen hoeveel tijd kan ik het doel behalen?

Één dag — X — *6 maanden*

Duurt het langer dan 6 maanden ? Dan kunt u het doel het beste opdelen in meerdere delen en het eerste deel hier beschrijven.:

____

Check 4: Is er iemand die ik om hulp kan vragen?

MIJN BROER LOOPT MEE

Check 5: Herinnering toevoegen in mijn agenda?

X JA ○ NEE

BELONING

Succes moet worden gevierd. Hoe beloont u zichzelf als u het doel heeft behaald?

Vul nu uw beloning in op het schema.

DE EERSTE STAP INVULLEN

In dit gedeelte gaat u de eerste stap formuleren in de richting van uw doel. Deze stap helpt u om na te denken over hoe u het doel het beste kunt inpassen in uw dagelijks leven.

Ga bij uzelf na:

- Wat is de allereerste stap die ik zou kunnen zetten om dit doel te bereiken?
- Wanneer wil ik deze stap hebben gezet?

Vul nu uw eerste stap in op het schema.

DRIE VOORBEELDEN VAN EERSTE STAPPEN ZIJN:

DE EERSTE STAP

Wat ga ik precies doen? IK GA EEN BLOKJE OM MET DE FIETS

Waar ga ik dit doen? IN MIJN BUURT

WANNEER GA IK DIT DOEN?

- ○ Vandaag
- ○ Morgen
- X Overmorgen
- ○ ANDERS:

DE EERSTE STAP

Wat ga ik precies doen? IK DAAG MIJZELF UIT OM EEN WANDELING VAN 5 KM TE MAKEN

Waar ga ik dit doen? IN DE OMGEVING VAN HET VRIJTHOF

WANNEER GA IK DIT DOEN?

- [ ] Vandaag
- [ ] Morgen
- [ ] Overmorgen
- [x] EIND VAN DE WEEK

DE EERSTE STAP

Wat ga ik precies doen? ONDANKS DAT IK HET SPANNEND VIND GA IK VANDAAG MET MIJN RUGZAK OVER ONGELIJK TERREIN LOPEN, ZODAT IK OP VAKANTIE DIE JUNGLE TOCHT KAN MAKEN

Waar ga ik dit doen? DOOR HET STADSPARK

WANNEER GA IK DIT DOEN?

- [x] Vandaag
- [ ] Morgen
- [ ] Overmorgen
- [ ] ANDERS:

OBSTAKELS EN ACTIES

Er zijn regelmatig obstakels in het spel die in de weg kunnen staan bij het zetten van de eerste stap. Als u hier vooraf over nadenkt en er alvast een actie bij verzint, dan komt u niet voor onverwachte tegenslagen te staan.

DRIE VOORBEELDEN VAN OBSTAKELS EN ACTIES ZIJN:

**OBSTAKEL** EEN HOOSBUI, TERWIJL IK ZOU GAAN WANDELEN	**ACTIE** IK PLAN DIRECT EEN VOLGEND MOMENT IN MIJN AGENDA
**OBSTAKEL** HET IS DRUK OP HET WERK, WAARDOOR IK VAAK MOET OVERWERKEN EN IK VERMOEID THUISKOM.	**ACTIE** IK START DE DAG MET EEN KORTE WANDELING, VOORDAT IK GA WERKEN.
**OBSTAKEL** IK VIND HET NIET LEUK OM ALLEEN TE SPORTEN. IK HEB DAN OP HET LAATSTE MOMENT GEEN ZIN.	**ACTIE** IK GA OP ZOEK NAAR EEN SPORTCLUB WAAR ER GEZAMENLIJK WORDT GESPORT OP MIJN NIVEAU

Voeg nu in het schema de belangrijkste obstakels toe, die horen bij uw eerste stap. Bedenk daarbij ook meteen een actie. Als u verwacht dat u geen last zult hebben van obstakels, dan kunt u dit veld overslaan.

Gefeliciteerd, u heeft uw eerste waardegerichte doelen schema ingevuld! Met behulp van de eerste stap kunt u binnenkort al aan de slag met uw doel. Als u de eerste stap heeft volbracht, dan kunt u de vervolgstappen gebruiken om steeds dichter bij uw doel te komen. De pagina's met vervolgstappen vindt u direct achter ieder schema in dit werkboek.

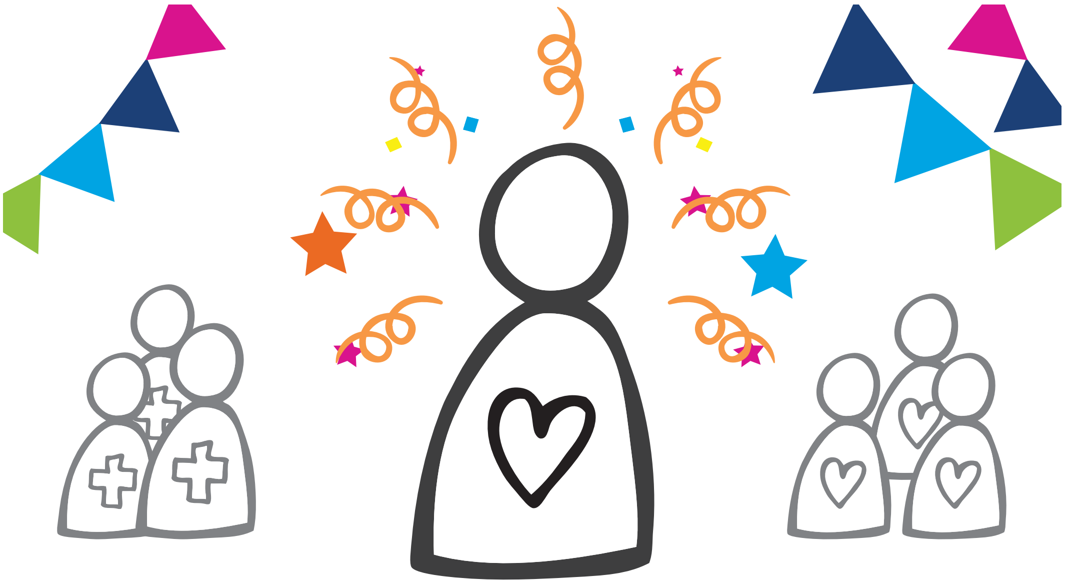

SCHEMA

Op de volgende pagina's vindt u twee volledig ingevulde schema's als voorbeeld. Heeft u alle schema's opgebruikt? Via **http://www.husite.nl/solace/formulieren** kunt u extra lege schema's downloaden en zelf uitprinten.

DE WAARDE zie pagina 13

ACTIEF ZIJN

HET DOEL zie pagina 21

OVER 4 MAANDEN LOOP IK MEE IN DE

AVONDVIERDAAGSE (5KM)

Check 1: Hoe graag wil ik dit doel behalen?

Als het doel niet zo belangrijk voor u is, dan is de kans dat het gaat lukken vaak een stuk kleiner.

Helemaal niet belangrijk — *ondergrens* — X — *Erg belangrijk*

Check 2: Hoe veel vertrouwen heb ik erin dat ik het kan?

Als u er veel vertrouwen in heeft, is de kans dat het gaat lukken vaak een stuk groter.

Helemaal geen vertrouwen — *ondergrens* — X — *Erg veel vertrouwen*

Check 3: Binnen hoeveel tijd kan ik het doel behalen?

Één dag — X — *6 maanden*

Duurt het langer dan 6 maanden ? Dan kunt u het doel het beste opdelen in meerdere delen en het eerste deel hier beschrijven.:

Check 4: Is er iemand die ik om hulp kan vragen?

MIJN BROER LOOPT MEE

Check 5: Herinnering toevoegen in mijn agenda?

X JA ○ NEE

BELONING zie pagina 23

UIT ETEN MET MIJN BROER

DE EERSTE STAP zie pagina 24

Wat ga ik precies doen? IK DAAG MIJZELF UIT OM EEN WANDELING VAN 5 KM TE MAKEN

Waar ga ik dit doen? IN DE OMGEVING VAN HET VRIJTHOF

WANNEER GA IK DIT DOEN?

- ○ Vandaag
- ○ Morgen
- ○ Overmorgen
- X ANDERS: EIND VAN DE WEEK

OBSTAKEL

REGEN

ACTIE IK MAG ÉÉN KEER IN DE WEEK OVERSLAAN, ANDERS REGENKLEDING

DE WAARDE zie pagina 13

VRIJHEID

HET DOEL zie pagina 21

IK WIL DRIE WEKEN EEN ACTIEVE REIS DOOR THAILAND MAKEN.

Check 1: Hoe graag wil ik dit doel behalen?

Als het doel niet zo belangrijk voor u is, dan is de kans dat het gaat lukken vaak een stuk kleiner.

Helemaal niet belangrijk — *ondergrens* — *Erg belangrijk* (X bij Erg belangrijk)

Check 2: Hoe veel vertrouwen heb ik erin dat ik het kan?

Als u er veel vertrouwen in heeft, is de kans dat het gaat lukken vaak een stuk groter.

Helemaal geen vertrouwen — *ondergrens* — X — *Erg veel vertrouwen*

Check 3: Binnen hoeveel tijd kan ik het doel behalen?

Één dag — X — *6 maanden*

Duurt het langer dan 6 maanden ? Dan kunt u het doel het beste opdelen in meerdere delen en het eerste deel hier beschrijven.:

Check 4: Is er iemand die ik om hulp kan vragen?

MIJN MAN GAAT MEE

Check 5: Herinnering toevoegen in mijn agenda?

X JA ○ NEE

BELONING zie pagina 23

VOOR HET EERST SINDS LANGE TIJD WEER OP VAKANTIE MET

MIJN MAN EN KINDEREN

DE EERSTE STAP zie pagina 24

Wat ga ik precies doen? ONDANKS DAT IK HET SPANNEND VIND GA IK

VANDAAG MET MIJN RUGZAK OVER ONGELIJK TERREIN LOPEN,

ZODAT IK OP VAKANTIE DIE JUNGLE TOCHT KAN MAKEN

Waar ga ik dit doen? DOOR HET STADSPARK

WANNEER GA IK DIT DOEN?

- [x] Vandaag
- [ ] Morgen
- [ ] Overmorgen
- [ ] ANDERS:

OBSTAKEL EEN HOOSBUI,

TERWIJL IK ZOU GAAN

WANDELEN

ACTIE IK PLAN DIRECT EEN

VOLGEND MOMENT IN MIJN

AGENDA WAAROP IK GA

WANDELEN.

DE WAARDE zie pagina 13

HET DOEL zie pagina 21

Check 1: Hoe graag wil ik dit doel behalen?

Als het doel niet zo belangrijk voor u is, dan is de kans dat het gaat lukken vaak een stuk kleiner.

Helemaal niet belangrijk — *ondergrens* — *Erg belangrijk*

Check 2: Hoe veel vertrouwen heb ik erin dat ik het kan?

Als u er veel vertrouwen in heeft, is de kans dat het gaat lukken vaak een stuk groter.

Helemaal geen vertrouwen — *ondergrens* — *Erg veel vertrouwen*

Check 3: Binnen hoeveel tijd kan ik het doel behalen?

Één dag — *6 maanden*

Duurt het langer dan 6 maanden ? Dan kunt u het doel het beste opdelen in meerdere delen en het eerste deel hier beschrijven.:

Check 4: Is er iemand die ik om hulp kan vragen?

Check 5: Herinnering toevoegen in mijn agenda?

JA NEE

BELONING zie pagina 23

DE EERSTE STAP zie pagina 24

Wat ga ik precies doen?

Waar ga ik dit doen?

WANNEER GA IK DIT DOEN?

- Vandaag
- Morgen
- Overmorgen
- ANDERS:

OBSTAKEL

ACTIE

DE VOLGENDE STAP

Wat ga ik precies doen?

Waar ga ik dit doen?

WANNEER GA IK DIT DOEN?

- Vandaag
- Morgen
- Overmorgen
- ANDERS:

OBSTAKEL

ACTIE

DE VOLGENDE STAP

Wat ga ik precies doen?

Waar ga ik dit doen?

WANNEER GA IK DIT DOEN?

- Vandaag
- Morgen
- Overmorgen
- ANDERS:

OBSTAKEL

ACTIE

DOEL BEHAALD?

- Ja, het doel is behaald.
  zie pagina 52
- Nee, het doel is niet de juiste keuze.
  zie pagina 53
- Nee, ik heb meer stappen nodig.
  Ga verder met het formuleren van de volgende stap vanaf pagina 55.

DE WAARDE zie pagina 13

HET DOEL zie pagina 21

Check 1: Hoe graag wil ik dit doel behalen?

Als het doel niet zo belangrijk voor u is, dan is de kans dat het gaat lukken vaak een stuk kleiner.

Helemaal niet belangrijk — *ondergrens* — *Erg belangrijk*

Check 2: Hoe veel vertrouwen heb ik erin dat ik het kan?

Als u er veel vertrouwen in heeft, is de kans dat het gaat lukken vaak een stuk groter.

Helemaal geen vertrouwen — *ondergrens* — *Erg veel vertrouwen*

Check 3: Binnen hoeveel tijd kan ik het doel behalen?

Één dag — *6 maanden*

Duurt het langer dan 6 maanden ? Dan kunt u het doel het beste opdelen in meerdere delen en het eerste deel hier beschrijven.:

Check 4: Is er iemand die ik om hulp kan vragen?

Check 5: Herinnering toevoegen in mijn agenda?

JA NEE

BELONING

zie pagina 23

DE EERSTE STAP

zie pagina 24

Wat ga ik precies doen?

Waar ga ik dit doen?

WANNEER GA IK DIT DOEN?

- Vandaag
- Morgen
- Overmorgen
- ANDERS:

OBSTAKEL

ACTIE

DE VOLGENDE STAP

Wat ga ik precies doen?

Waar ga ik dit doen?

WANNEER GA IK DIT DOEN?

- Vandaag
- Morgen
- Overmorgen
- ANDERS:

OBSTAKEL

ACTIE

DE VOLGENDE STAP

Wat ga ik precies doen?

Waar ga ik dit doen?

WANNEER GA IK DIT DOEN?

- Vandaag
- Morgen
- Overmorgen
- ANDERS:

OBSTAKEL

ACTIE

DOEL BEHAALD?

- Ja, het doel is behaald.
  zie pagina 52
- Nee, het doel is niet de juiste keuze.
  zie pagina 53
- Nee, ik heb meer stappen nodig.
  Ga verder met het formuleren van de volgende stap vanaf pagina 55.

DE WAARDE zie pagina 13

HET DOEL zie pagina 21

Check 1: Hoe graag wil ik dit doel behalen?

Als het doel niet zo belangrijk voor u is, dan is de kans dat het gaat lukken vaak een stuk kleiner.

Helemaal niet belangrijk — *ondergrens* — *Erg belangrijk*

Check 2: Hoe veel vertrouwen heb ik erin dat ik het kan?

Als u er veel vertrouwen in heeft, is de kans dat het gaat lukken vaak een stuk groter.

Helemaal geen vertrouwen — *ondergrens* — *Erg veel vertrouwen*

Check 3: Binnen hoeveel tijd kan ik het doel behalen?

Één dag — *6 maanden*

Duurt het langer dan 6 maanden ? Dan kunt u het doel het beste opdelen in meerdere delen en het eerste deel hier beschrijven.:

Check 4: Is er iemand die ik om hulp kan vragen?

Check 5: Herinnering toevoegen in mijn agenda?

- JA
- NEE

BELONING

zie pagina 23

DE EERSTE STAP

zie pagina 24

Wat ga ik precies doen?

Waar ga ik dit doen?

WANNEER GA IK DIT DOEN?

- Vandaag
- Morgen
- Overmorgen
- ANDERS:

OBSTAKEL

ACTIE

DE VOLGENDE STAP

Wat ga ik precies doen?

Waar ga ik dit doen?

WANNEER GA IK DIT DOEN?

- Vandaag
- Morgen
- Overmorgen
- ANDERS:

OBSTAKEL

ACTIE

DE VOLGENDE STAP

Wat ga ik precies doen?

Waar ga ik dit doen?

WANNEER GA IK DIT DOEN?

- Vandaag
- Morgen
- Overmorgen
- ANDERS:

**OBSTAKEL**	**ACTIE**

DOEL BEHAALD?

- Ja, het doel is behaald.
  zie pagina 52
- Nee, het doel is niet de juiste keuze.
  zie pagina 53
- Nee, ik heb meer stappen nodig.
  Ga verder met het formuleren van de volgende stap vanaf pagina 55.

DE WAARDE zie pagina 13

HET DOEL zie pagina 21

Check 1: Hoe graag wil ik dit doel behalen?

Als het doel niet zo belangrijk voor u is, dan is de kans dat het gaat lukken vaak een stuk kleiner.

Helemaal niet belangrijk — *ondergrens* — *Erg belangrijk*

Check 2: Hoe veel vertrouwen heb ik erin dat ik het kan?

Als u er veel vertrouwen in heeft, is de kans dat het gaat lukken vaak een stuk groter.

Helemaal geen vertrouwen — *ondergrens* — *Erg veel vertrouwen*

Check 3: Binnen hoeveel tijd kan ik het doel behalen?

Één dag — *6 maanden*

Duurt het langer dan 6 maanden ? Dan kunt u het doel het beste opdelen in meerdere delen en het eerste deel hier beschrijven.:

Check 4: Is er iemand die ik om hulp kan vragen?

Check 5: Herinnering toevoegen in mijn agenda?

○ JA ○ NEE

BELONING

zie pagina 23

DE EERSTE STAP

zie pagina 24

Wat ga ik precies doen?

Waar ga ik dit doen?

WANNEER GA IK DIT DOEN?

- Vandaag
- Morgen
- Overmorgen
- ANDERS:

OBSTAKEL

ACTIE

DE VOLGENDE STAP

Wat ga ik precies doen?

Waar ga ik dit doen?

WANNEER GA IK DIT DOEN?

- Vandaag
- Morgen
- Overmorgen
- ANDERS:

OBSTAKEL

ACTIE

DE VOLGENDE STAP

Wat ga ik precies doen?

Waar ga ik dit doen?

WANNEER GA IK DIT DOEN?

- Vandaag
- Morgen
- Overmorgen
- ANDERS:

OBSTAKEL

ACTIE

DOEL BEHAALD?

- Ja, het doel is behaald.
  zie pagina 52
- Nee, het doel is niet de juiste keuze.
  zie pagina 53
- Nee, ik heb meer stappen nodig.
  Ga verder met het formuleren van de volgende stap vanaf pagina 55.

DE WAARDE

zie pagina 13

HET DOEL

zie pagina 21

Check 1: Hoe graag wil ik dit doel behalen?

Als het doel niet zo belangrijk voor u is, dan is de kans dat het gaat lukken vaak een stuk kleiner.

Helemaal niet belangrijk — *ondergrens* — *Erg belangrijk*

Check 2: Hoe veel vertrouwen heb ik erin dat ik het kan?

Als u er veel vertrouwen in heeft, is de kans dat het gaat lukken vaak een stuk groter.

Helemaal geen vertrouwen — *ondergrens* — *Erg veel vertrouwen*

Check 3: Binnen hoeveel tijd kan ik het doel behalen?

Één dag — *6 maanden*

Duurt het langer dan 6 maanden ? Dan kunt u het doel het beste opdelen in meerdere delen en het eerste deel hier beschrijven.:

Check 4: Is er iemand die ik om hulp kan vragen?

Check 5: Herinnering toevoegen in mijn agenda?

- JA
- NEE

BELONING zie pagina 23

DE EERSTE STAP zie pagina 24

Wat ga ik precies doen?

Waar ga ik dit doen?

WANNEER GA IK DIT DOEN?

- Vandaag
- Morgen
- Overmorgen
- ANDERS:

OBSTAKEL

ACTIE

DE VOLGENDE STAP

Wat ga ik precies doen?

Waar ga ik dit doen?

WANNEER GA IK DIT DOEN?

- Vandaag
- Morgen
- Overmorgen
- ANDERS:

OBSTAKEL

ACTIE

DE VOLGENDE STAP

Wat ga ik precies doen?

Waar ga ik dit doen?

WANNEER GA IK DIT DOEN?

- Vandaag
- Morgen
- Overmorgen
- ANDERS:

OBSTAKEL

ACTIE

DOEL BEHAALD?

- Ja, het doel is behaald.
  zie pagina 52
- Nee, het doel is niet de juiste keuze.
  zie pagina 53
- Nee, ik heb meer stappen nodig.
  Ga verder met het formuleren van de volgende stap vanaf pagina 55.

DOEL BEHAALD

Gefeliciteerd! U heeft alle noodzakelijke stappen doorlopen om het doel te bereiken. Hopelijk heeft dit doel u dichter bij uw waarde gebracht. **Vergeet uw zelfgekozen beloning niet!**

TERUGKIJKEN EN VOORUIT KIJKEN

Welke vaardigheden of kwaliteiten hebben u goed geholpen bij uw doel? Wellicht kunt u dit opnieuw toepassen bij een volgend doel.

Notitieruimte:

Had u vooraf goed ingeschat hoe wenselijk en haalbaar het doel was? Heeft u het doel bereikt binnen de vooraf ingeschatte tijd? Probeer uw ervaring te gebruiken bij het inschatten van de wenselijkheid en haalbaarheid van een volgend doel.

Notitieruimte:

Tijd voor een nieuw doel?

Gaat u een vervolgdoel stellen? Gebruik een nieuw schema voor dit nieuwe doel en probeer dit in te vullen met bovenstaande vragen in gedachten.

HET DOEL IS NIET DE JUISTE KEUZE.

Soms is het niet langer wenselijk om het doel te behalen. Het kan zijn dat het doel **lastiger** blijkt dan gedacht, of dat u uw energie liever aan **iets anders** besteedt. Ook kan het zijn dat het doel andere belangrijke doelen in de weg zit.

Lukt het niet om uw doel te behalen dan kunt u uzelf de volgende vragen stellen:

- Is er iemand in mijn omgeving die mij kan helpen bij het behalen van dit doel?
- Heb ik alle mogelijke obstakels opgeschreven en heb ik manieren gevonden om hier goed mee om te gaan?

Soms kan het helpen om hier nog een keer over na te denken en een nieuw plan te maken. Mochten deze vragen niet helpen, dan kunt u ook stoppen met proberen dit doel te behalen en teruggaan naar de basis: **uw waarde**.

Met behulp van deze waarde kunt u een alternatief doel kiezen dat beter aansluit bij uw dagelijks leven. Vanaf pagina 21 vindt u de instructie voor het kiezen van een waardegericht doel.

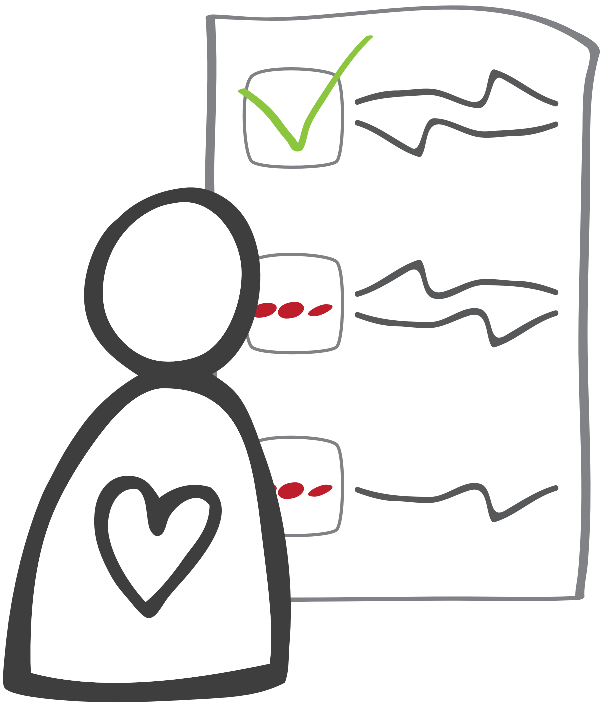

DE VOLGENDE STAP

Wat ga ik precies doen?

Waar ga ik dit doen?

WANNEER GA IK DIT DOEN?

- Vandaag
- Morgen
- Overmorgen
- ANDERS:

OBSTAKEL

ACTIE

DE VOLGENDE STAP

Wat ga ik precies doen?

Waar ga ik dit doen?

WANNEER GA IK DIT DOEN?

- Vandaag
- Morgen
- Overmorgen
- ANDERS:

OBSTAKEL

ACTIE

DE VOLGENDE STAP

Wat ga ik precies doen?

Waar ga ik dit doen?

WANNEER GA IK DIT DOEN?

- Vandaag
- Morgen
- Overmorgen
- ANDERS:

OBSTAKEL

ACTIE

DE VOLGENDE STAP

Wat ga ik precies doen?

Waar ga ik dit doen?

WANNEER GA IK DIT DOEN?

- Vandaag
- Morgen
- Overmorgen
- ANDERS:

OBSTAKEL

ACTIE

DE VOLGENDE STAP

Wat ga ik precies doen?

Waar ga ik dit doen?

WANNEER GA IK DIT DOEN?

- Vandaag
- Morgen
- Overmorgen
- ANDERS:

OBSTAKEL

ACTIE

DE VOLGENDE STAP

Wat ga ik precies doen?

Waar ga ik dit doen?

WANNEER GA IK DIT DOEN?

- Vandaag
- Morgen
- Overmorgen
- ANDERS:

OBSTAKEL

ACTIE

DE VOLGENDE STAP

Wat ga ik precies doen?

Waar ga ik dit doen?

WANNEER GA IK DIT DOEN?

- Vandaag
- Morgen
- Overmorgen
- ANDERS:

**OBSTAKEL**	**ACTIE**

DE VOLGENDE STAP

Wat ga ik precies doen?

Waar ga ik dit doen?

WANNEER GA IK DIT DOEN?

- Vandaag
- Morgen
- Overmorgen
- ANDERS:

OBSTAKEL

ACTIE

DE VOLGENDE STAP

Wat ga ik precies doen?

Waar ga ik dit doen?

WANNEER GA IK DIT DOEN?

- Vandaag
- Morgen
- Overmorgen
- ANDERS:

OBSTAKEL

ACTIE

DO IT YOURSELF

Er gebeurt veel tijdens de revalidatie. U krijgt dagelijks nieuwe informatie en u gaat aan de slag met verschillende behandelaren. Sommige ervaringen tijdens de revalidatie zullen voor u **extra** belangrijk zijn. Bijvoorbeeld:

1. U herkent een situatie die tijdens de voorlichting wordt besproken.
2. U krijgt een belangrijk advies of inzicht tijdens de behandeling.

Met behulp van deze oefening kunt u de momenten die u belangrijk vindt **opslaan** en **bewaren**, zodat deze momenten u ook na afloop van de revalidatie kunnen **ondersteunen**.

KORTE HANDLEIDING

1. Kies een **belangrijk moment** uit de revalidatie
2. Sla het moment op met behulp van een **geheugensteun**
3. Gebruik een overzicht van deze momenten als **naslagwerk**, **gesprekshulpmiddel** of **herinnering** in uw dagelijkse omgeving.

1. KIES EEN MOMENT

Stel uzelf iedere week de volgende vragen:

- Wat zijn de belangrijkste inzichten en succeservaringen tijdens de pijn-revalidatie geweest?
- Welke momenten helpen mij om een betekenisvol leven te leiden, met de pijn?
- Zijn er adviezen of gesprekken geweest waar ik iets aan heb gehad?
- Welke vaardigheden heb ik aangeleerd, welke kwaliteiten en mogelijkheden heb ik ontdekt?

Alle antwoorden op deze vragen zijn momenten die u kunt opslaan!

DRIE VOORBEELDEN VAN BELANGRIJKE MOMENTEN ZIJN:

WEEK 01

* INZICHT: ALS IK PIJN HEB, HOEFT DAT NIET TE BETEKENEN DAT ER IETS KAPOT IS!

* IK HEB EEN GOED GESPREK MET MIJN PARTNER GEHAD. ZIJ IS NU MINDER BEZORGD ALS IK GA LOPEN

* IK HEB TOCH DE TRAP GENOMEN, IN PLAATS VAN DE LIFT, TERWIJL IK EEN SLECHTE DAG HAD.

Vanaf pagina 70 heeft u voor iedere week ruimte om belangrijke momenten te noteren.

2. SLA HET MOMENT OP

Sla alle moment op die u wilt blijven onthouden, met behulp van de momentkaart. Dit gaat als volgt:

1. Schrijf het moment in steekwoorden op de bovenste helft van de moment kaart

2. De onderste helft van de kaart geeft ruimte voor een geheugensteun, zoals een foto, tekening, liedtekst, of motto. Als u een geheugensteun toevoegt, dan is de herinnering aan het moment gemakkelijker op te roepen.

3. Sommige geheugensteunen (bijvoorbeeld een lied of poster) zijn niet op de kaart te plakken. Omschrijf dan op de onderste helft welke geheugensteun u gaat gebruiken.

DRIE VOORBEELDEN VAN MOMENTENKAARTEN ZIJN:

MOMENT IK HAD EEN SLECHTE DAG EN IK WILDE EIGENLIJK NIET OEFENEN. TOEN IK AANKWAM HEB IK ER TOCH VOOR GEKOZEN OM DE TRAP TE NEMEN, DIT GAF ME VERTROUWEN.

GEHEUGENSTEUN

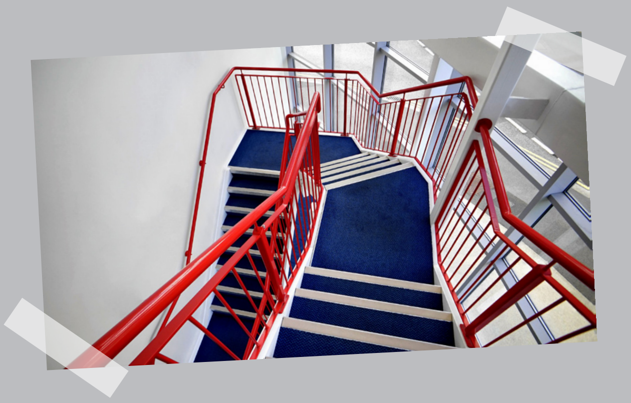

MOMENT EROVER PRATEN HELPT. MIJN PARTNER IS NU MINDER BEZORGD ALS IK GA LOPEN.

GEHEUGENSTEUN

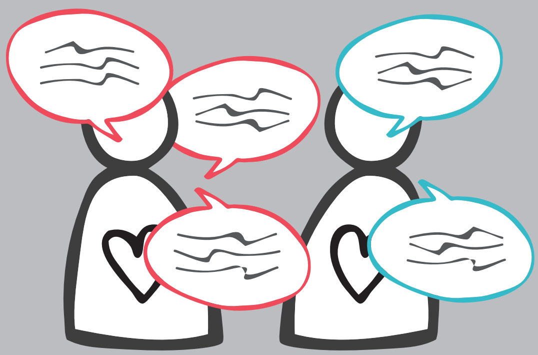

MOMENT ALS IK PIJN HEB, HOEFT DAT NIET TE BETEKENEN DAT ER IETS KAPOT IS

GEHEUGENSTEUN

PIJN ≠ SCHADE

STEL UZELF IEDERE WEEK DE VOLGENDE VRAGEN:

- Wat zijn de belangrijkste inzichten en succeservaringen tijdens de pijn-revalidatie geweest?
- Welke momenten helpen mij om een betekenisvol leven te leiden, met de pijn?
- Zijn er adviezen of gesprekken geweest waar ik iets aan heb gehad?
- Welke vaardigheden heb ik aangeleerd, welke kwaliteiten en mogelijkheden heb ik ontdekt?

WEEK 01

WEEK 02

WEEK 03

WEEK 04

WEEK 05

STEL UZELF IEDERE WEEK DE VOLGENDE VRAGEN:

Wat zijn de belangrijkste inzichten en succeservaringen tijdens de pijn-revalidatie geweest?
Welke momenten helpen mij om een betekenisvol leven te leiden, met de pijn?
Zijn er adviezen of gesprekken geweest waar ik iets aan heb gehad?
Welke vaardigheden heb ik aangeleerd, welke kwaliteiten en mogelijkheden heb ik ontdekt?

WEEK 06

WEEK 07

WEEK 08

WEEK 09

WEEK 10

STEL UZELF IEDERE WEEK DE VOLGENDE VRAGEN:

Wat zijn de belangrijkste inzichten en succeservaringen tijdens de pijn-revalidatie geweest?
Welke momenten helpen mij om een betekenisvol leven te leiden, met de pijn?
Zijn er adviezen of gesprekken geweest waar ik iets aan heb gehad?
Welke vaardigheden heb ik aangeleerd, welke kwaliteiten en mogelijkheden heb ik ontdekt?

WEEK 11

WEEK 12

WEEK 13

WEEK 14

WEEK 15

3. GEBRUIK DE MOMENTEN

Als u tijdens de revalidatie regelmatig momenten bewaart, heeft u aan het eind van de revalidatie een verzameling van alle ervaringen die voor u belangrijk zijn. Deze momenten kunt u als volgt gebruiken:

- **Naslagwerk**: Het kan zijn dat u op een bepaald moment na de revalidatie behoefte heeft terug te lezen welke ervaringen u heeft gehad.

- **Gespreksmiddel**: Niet iedereen begrijpt hoe het is om te leven met de gevolgen van chronische pijn. Vaak zorgt een gebrek aan informatie ervoor dat uw omgeving niet altijd even behulpzaam reageert, of de steun kan bieden die u nodig heeft. Als mensen in uw omgeving beter weten wat er aan de hand is, dan is de kans groot dat u zich beter gesteund voelt. Met behulp van deze momentkaarten kunt u beter uitleggen wat u tijdens de revalidatie uitvoert.

- **Geheugensteun**: Een geheugensteun kan goed van pas komen bij het onthouden van een moment uit de behandeling. Als u bijvoorbeeld trots bent op uw vooruitgang, dan kan het u helpen om daar regelmatig aan terug te denken. De geheugensteun kan u hierbij ondersteunen. Plaats de geheugensteun op een zichtbare plaats in uw omgeving. Als u de geheugensteun later opmerkt, dan denkt u automatisch terug aan de bijbehorende ervaring.

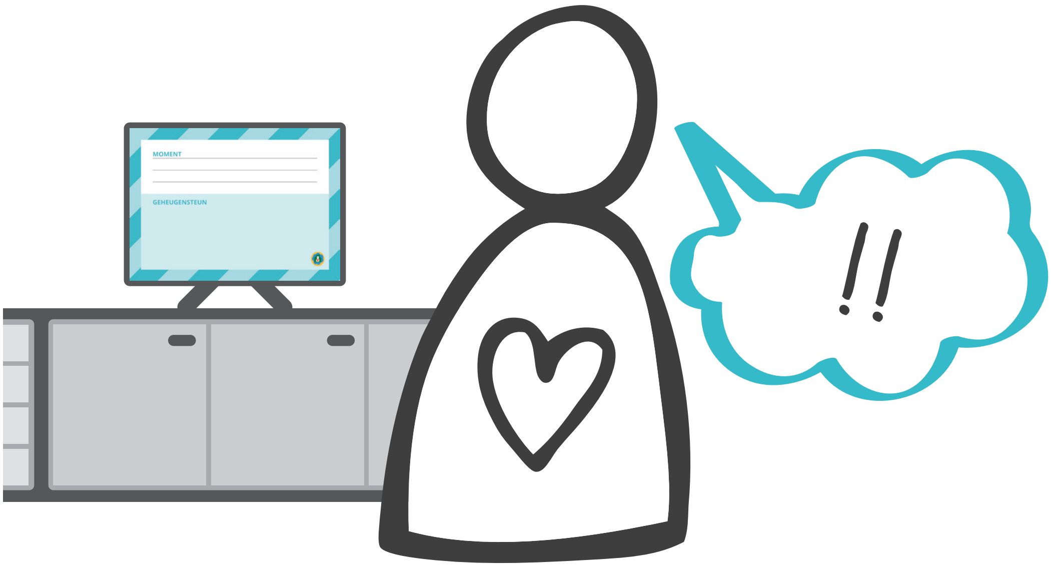

EXTRA: NA DE BEHANDELING

Wanneer u door alle opgeslagen momenten bladert en terugkijkt op de revalidatie, welke momenten zijn dan voor u het belangrijkst geweest? Aan welke momenten verwacht u ook in de komende jaren nog steun te hebben?

Notitieruimte:

Zijn er belangrijke ervaringen van de revalidatieperiode die nog niet zijn opgeslagen in momenten? Misschien kunt u deze nu nog toevoegen?

Notitieruimte:

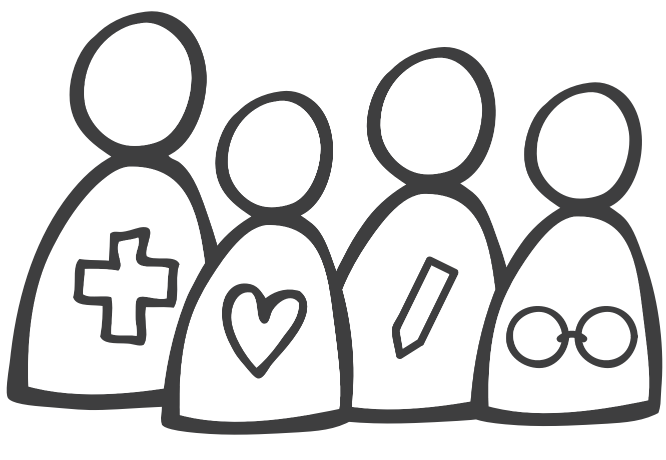

Supplement: Multimedia Appendix 4 [file humanfactors_v7i4e21545_app4.pdf]
