# Supplementary material for: Exploring the Feasibility of Relapse Prevention Strategies in Interdisciplinary Multimodal Pain Therapy Programs: Qualitative Study
Source: JMIR Hum Factors. 2020 Dec 11;7(4):e21545. doi: 10.2196/21545 (PMC7762683; doi:10.2196/21545)
Supplement: Multimedia Appendix 5 [file humanfactors_v7i4e21545_app5.pdf]

SUPPLEMENT 3

TRANSLATED TOPIC LIST FOR FOCUS GROUP.

Topic list for Focus Group.

General procedure:

- The moderator asks a question.
- All participants individually write down their responses and associations on post-its.
- Participants discuss their input.
- The moderator provides a concluding summary and proceeds to the next question.

Introduction question

1 During this session, we are mainly interested in your experiences concerning the feasibility of the prototype workbook. If you reflect on the first moment that you browsed through the workbook, what was your first impression?

Key Questions

Rationale:

- To evaluate the content and action mechanisms of both intervention (question 1-3).
- To evaluate the form of the current prototype (question 4).
- To evaluate the procedure of using the workbook in the context of the treatment program (question 5-7).

1 What are your positive experiences with each of the two interventions that are present in the workbook?

2 What are your negative experiences with each of the two interventions that are present in the workbook?

3 Overlooking the previously discussed negative experiences, what components or aspects of the workbook would you like to have improved?

4 We decided to offer the current prototype interventions in the form of a workbook.
What would you like to change with respect to the form and layout of the current prototype? (Optional probing suggestions: deleting or adding specific components? Are there more suitable options to present the interventions? Are there parts of the interventions that you would like to have presented in a different way?).

5 During which moments *during* the treatment did you use the workbook? What went well and what could be improved?

6 How would you like to use the workbook in an ideal situation *during* the treatment program? (optional: discuss together with question 5)

7 How would you like to have the patients use the workbook *after* the treatment program has ended?

- **After summarizing each key question**: Is this a complete and accurate summary of the most important themes during this discussion?

Concluding questions

1	If you look back on everything that has been discussed today, what is the most important theme that you would like to emphasize with respect to the future development of the prototype workbook?
2	Did we miss important themes or topics that are important for future development?
